# X-linked genes influence various complex traits in dairy cattle

**DOI:** 10.1186/s12864-023-09438-7

**Published:** 2023-06-19

**Authors:** Marie-Pierre Sanchez, Clémentine Escouflaire, Aurélia Baur, Fiona Bottin, Chris Hozé, Mekki Boussaha, Sébastien Fritz, Aurélien Capitan, Didier Boichard

**Affiliations:** 1 Université Paris-Saclay, INRAE, AgroParisTech, GABI, Jouy-en-Josas, 78350 France; 2 Eliance, Paris, 75012 France

**Keywords:** Dairy cattle, X chromosome, GWAS, Meta-analyses

## Abstract

**Background:**

The search for quantitative trait loci (QTL) affecting traits of interest in mammals is frequently limited to autosomes, with the X chromosome excluded because of its hemizygosity in males. This study aimed to assess the importance of the X chromosome in the genetic determinism of 11 complex traits related to milk production, milk composition, mastitis resistance, fertility, and stature in 236,496 cows from three major French dairy breeds (Holstein, Montbéliarde, and Normande) and three breeds of regional importance (Abondance, Tarentaise, and Vosgienne).

**Results:**

Estimates of the proportions of heritability due to autosomes and X chromosome (h²_X_) were consistent among breeds. On average over the 11 traits, h²_X_=0.008 and the X chromosome explained ~ 3.5% of total genetic variance. GWAS was performed within-breed at the sequence level (~ 200,000 genetic variants) and then combined in a meta-analysis. QTL were identified for most breeds and traits analyzed, with the exception of Tarentaise and Vosgienne and two fertility traits. Overall, 3, 74, 59, and 71 QTL were identified in Abondance, Montbéliarde, Normande, and Holstein, respectively, and most were associated with the most-heritable traits (milk traits and stature). The meta-analyses, which assessed a total of 157 QTL for the different traits, highlighted new QTL and refined the positions of some QTL found in the within-breed analyses. Altogether, our analyses identified a number of functional candidate genes, with the most notable being *GPC3*, *MBNL3*, *HS6ST2*, and *DMD* for dairy traits; *TMEM164*, *ACSL4*, *ENOX2*, *HTR2C*, *AMOT*, and *IRAK1* for udder health; *MAMLD1* and *COL4A6* for fertility; and *NRK*, *ESX1*, *GPR50*, *GPC3*, and *GPC4* for stature.

**Conclusions:**

This study demonstrates the importance of the X chromosome in the genetic determinism of complex traits in dairy cattle and highlights new functional candidate genes and variants for these traits. These results could potentially be extended to other species as many X-linked genes are shared among mammals.

**Supplementary Information:**

The online version contains supplementary material available at 10.1186/s12864-023-09438-7.

## Background

Since the late 2000s, significant efforts have been made to decipher the genetic determinism of complex traits using genome-wide association studies (GWAS) or meta-analyses of GWAS results in different species, in particular in humans [1]. To date, the largest GWAS have focused on human height, which is a highly heritable and easily measured trait and thus one for which very large datasets are available [2, 3]. Despite all these efforts, in all human GWAS results published so far—including a very recent study that examined human height in more than five million individuals [3]—a portion of the heritability of the studied traits remains unexplained [4, 5]. The percentage differs among studies, but even small amounts of unexplained variability can be detrimental to our understanding of the biological mechanisms underlying traits of interest. Furthermore, a lack of information on some of the genetic variants associated with complex traits, such as some diseases for example, can decrease the accuracy of predictions of genetic risk at the population or individual level [6].

In livestock species, the bovine genome was one of the first to be sequenced, in 2009 [7]. As in humans, considerable efforts have been made in the last decade to discover the genes and genomic variants that are involved in the genetic determinism of various complex traits of interest [8, 9], including stature, for which a large-scale meta-analysis was conducted [10] as part of the 1000 Bull Genomes project [11]. One of the expected benefits in cattle, and in livestock species in general, is improving the prediction of complex traits for breeding programs [12].

However, in most GWAS conducted in mammals, and in particular in the large-scale GWAS of stature in cattle [10] and humans [13], the focus has mainly been on autosomes, with the X chromosome often excluded due to its unique mode of inheritance. Genetic studies of the X chromosome are complicated by two factors: male hemizygosity (XY) in the non-pseudoautosomal region (non-PAR), which covers the majority of the X chromosome in mammals [14, 15], and dosage compensation, i.e., the inactivation of one X chromosome, in XX females during early development, which ensures equivalent X-linked gene expression in cells of animals of both sexes [16]. Although it has been much less studied than in humans or model species, the phenomenon of dosage compensation has also been demonstrated in cattle [17]. Consequently, in order to include the X chromosome in a genetic study, researchers must use different or separate treatments for bulls and cows and make assumptions about dosage compensation [18, 19], which is rarely done. However, the X chromosome is the second-largest chromosome in the bovine genome and contains 1132 annotated genes (Ensembl release 107 - Jan 2023 [20]), i.e., more than 4% of all annotated genes in the entire genome, with most (1098) located in the non-PAR region. Therefore, the exclusion of the X chromosome from efforts to decipher the genetic determinism of complex traits or to improve the breeding values of animals may mean that a significant number of relevant genes are missed, and may result in a loss of efficiency in genomic selection [19].

In France, programs of genomic selection have been active since 2009 for the national dairy breeds Montbéliarde, Normande, and Holstein [21], and since 2016 for the regional dairy breeds Abondance, Tarentaise, and Vosgienne [22]. As a result, data are available for several thousand to several hundred thousand cows in these breeds, including phenotypes for various complex traits of economic interest as well as genotypes from the bovine EuroGMD chip [23]. Although X-chromosome variants have not yet been incorporated into genomic prediction equations for estimating the breeding values of animals, more than 1000 SNPs located in this region are included on the bovine Illumina EuroGMD chip. Here, we leveraged this large dataset to assess the relative importance of the X chromosome in the genetic determinism of complex traits in dairy cattle and to identify candidate causative X-linked genes and variants.

Specifically, we investigated 11 traits related to milk production, mastitis resistance, fertility, and stature that were measured in 236,496 cows of the six breeds (Montbéliarde, Normande, Holstein, Abondance, Tarentaise, and Vosgienne), with three objectives: [1] to estimate the respective proportions of heritability due to autosomes and the X chromosome in each breed and for each trait, [2] to perform within-breed association analyses that evaluated the effects of X-chromosome variants on each trait using genotypes imputed at the sequence level, and [3] for each trait, to combine the results obtained in the six different breeds in a meta-analysis.

## Results

In the six breeds examined here— Montbéliarde, Normande, Holstein, Abondance, Tarentaise, and Vosgienne —a number of traits are routinely measured for breeding, including traits related to milk production and composition (milk yield (MY), protein yield (PY), fat yield (FY), protein content (PC), and fat content (FC)), udder health (clinical mastitis (MAST) and somatic cell score (SCS)), fertility (interval between calving and first fertilizing artificial insemination (ICFI), heifers’ conception rate (HCR), and lactating cows’ conception rate (CCR)), and stature (STAT). To complement these phenotypes, genotypes are available for a subset of cows, typically based on one of several medium-density SNP chips used in the last 12 years, especially the Illumina EuroGMD chip. The number of cows for which both phenotypes and genotypes are available varies greatly among breeds, from 2555 cows in Vosgienne to 81,815 cows in Holstein (Table 1). Note that, due to computational limitations, we restricted the sample size of the two largest breeds by randomly sampling 61,881 Montbéliarde and 81,815 Holstein cows from the available 162,419 Montbéliarde and 315,674 Holstein cows with both phenotypes and genotypes.

**Table 1:** Number of cows with both genotypes and phenotypes per trait and breed

Trait	Abbr.	Abondance	Tarentaise	Vosgienne	Montbéliarde	Normande	Holstein
Milk yield	MY	6311	3326	2478	61,881	62,629	81,815
Protein yield	PY	6311	3326	2477	61,881	62,619	81,815
Fat yield	FY	6310	3326	2475	61,881	62,612	81,815
Protein content	PC	6310	3326	2475	61,881	62,615	81,815
Fat content	FC	6306	3326	2473	61,881	62,600	81,815
Somatic cell score	SCS	6703	3615	2555	61,881	65,105	81,815
Clinical mastitis	MAST	5411	2989	0	61,881	42,859	81,815
Interval between calving and first artificial insemination	ICFI	5845	2926	2284	61,881	57,012	81,815
Heifers’ conception rate	HCR	5868	3402	2297	61,881	69,695	81,815
Lactating cows’ conception rate	CCR	5067	2707	2216	61,881	52,816	81,815
Stature	STAT	4782	2887	2013	61,881	39,872	81,815

### Heritability due to autosomes and X chromosome

To estimate heritabilities due to autosomes (h²_AUT_) and the X chromosome (h²_X_), we first used a restricted maximum likelihood (REML) approach that included two random polygenic effects estimated using genomic relationship matrices (GRM), which were derived from 53,469 autosomal and 1147 X-linked SNPs, respectively. In all breeds, the most heritable traits were STAT (0.42–0.72) and milk composition traits, i.e., PC (0.52–0.75) and FC (0.52–0.72), while MAST and fertility traits (ICFI, HCR, and CCR) were the least heritable (0.01–0.09). SCS, MY, PY, and FY presented intermediate heritability, with h² estimates ranging from 0.22 to 0.40 (Table 2). For all breeds and traits, h²_AUT_ ranged from 0.008 (ICFI in Vosgienne) to 0.74 (PC in Vosgienne), while h²_X_ ranged from 0.000 (MAST, HCR, and/or CCR in multiple breeds; STAT in Tarentaise) to 0.04 (PC in Tarentaise). For the most-heritable traits, h² was mainly explained by autosomes; instead, the proportion of genetic variance explained by the X chromosome was higher for SCS, HCR, and CCR in Tarentaise and for ICFI in Vosgienne. The standard error of estimates ranged from 0.002 to 0.035 for h²_AUT_ and from 0.0004 to 0.019 for h²_X_. On average across all breeds, around 3.5% of the total genetic variance of each trait was explained by the X chromosome.

**Table 2:** Overall heritability (h²) and heritability due to autosomes (h²_AUT_) and X chromosome (h²_X_)^1^

	ABO			Tarentaise			Vosgienne			Montbéliarde			Normande			Holstein		
	h²	h²_AUT_	h²_X_	h²	h²_AUT_	h²_X_	h²	h²_AUT_	h²_X_	h²	h²_AUT_	h²_X_	h²	h²_AUT_	h²_X_	h²	h²_AUT_	h²_X_
MY	0.324	0.315	0.009	0.354	0.340	0.015	0.398	0.371	0.027	0.345	0.338	0.007	0.260	0.253	0.007	0.340	0.330	0.006
PY	0.260	0.251	0.009	0.316	0.304	0.012	0.345	0.325	0.020	0.285	0.279	0.006	0.224	0.217	0.007	0.256	0.249	0.007
FY	0.299	0.289	0.010	0.384	0.365	0.019	0.356	0.333	0.023	0.324	0.317	0.007	0.228	0.222	0.006	0.306	0.299	0.007
PC	0.707	0.678	0.029	0.728	0.687	0.041	0.747	0.738	0.009	0.652	0.636	0.016	0.522	0.515	0.007	0.660	0.650	0.014
FC	0.677	0.668	0.010	0.719	0.698	0.020	0.723	0.712	0.011	0.630	0.619	0.011	0.522	0.513	0.009	0.660	0.644	0.012
SCS	0.223	0.218	0.005	0.209	0.185	0.024	0.219	0.218	0.001	0.227	0.223	0.004	0.180	0.175	0.005	0.240	0.235	0.008
MAST	0.024	0.023	0.001	0.021	0.021	0.000	-	-	-	0.027	0.027	0.000	0.033	0.033	0.000	0.035	0.034	0.001
ICFI	0.049	0.048	0.001	0.055	0.054	0.002	0.019	0.008	0.011	0.080	0.077	0.004	0.070	0.064	0.005	0.094	0.093	0.002
HCR	0.035	0.033	0.002	0.023	0.019	0.004	0.043	0.043	0.000	0.018	0.016	0.001	0.020	0.019	0.000	0.010	0.012	0.000
CCR	0.038	0.038	0.000	0.051	0.025	0.027	0.043	0.043	0.000	0.041	0.041	0.001	0.049	0.048	0.001	0.070	0.068	0.001
STAT	0.641	0.639	0.002	0.704	0.704	0.000	0.715	0.706	0.010	0.564	0.554	0.010	0.559	0.543	0.015	0.420	0.414	0.008

^1^ Standard errors ranged from 0.002 to 0.035 for h²_AUT_ and from 0.0004 to 0.019 for h²_X_

### Within-breed linkage disequilibrium on X chromosome

Linkage disequilibrium data, calculated for chromosomes 2 and X in the 6 breeds, revealed an expected decline in r^2^ as the distance between markers increased (Fig. S1). Remarkably, the X chromosome generally exhibited higher linkage disequilibrium levels irrespective of marker distance. Moreover, regional breeds (Abondance, Tarentaise, and Vosgienne) exhibited higher levels of linkage disequilibrium, consistent with their lower effective population size.

### Within-breed and meta-analyses of association

For all cows with both genotypes and phenotypes, sequence-level genotypes were imputed from the 50k EuroGMD genotypes via an intermediate HD density step; details on the imputation procedure can be found in the Materials and Methods. A multibreed sample of 2712 sequenced bulls, which included between 4 (Vosgienne) and 1019 (Holstein) bulls of each of the six breeds, was used as a reference for sequence-level imputation. After we removed variants with a MAF lower than 0.005 and an imputation accuracy lower than 0.2 (as assessed by the R squared (R²) value generated by Minimac software [24]), the mean imputation accuracy ranged from 0.69 for Abondance to 0.82 for Normande (Table 3).

**Table 3:** Features of populations and sequence variants analyzed

Breed	# 50k genotypes	# HD genotypes	# WGS animals^2^	# variants after filtering^1^	Mean^1^ imputation R²	Mean^1^ MAF
Abondance	7449	199	9	154,966	0.69	0.18
Tarentaise	3969	179	12	181,473	0.79	0.19
Vosgienne	2910	181	4	170,560	0.77	0.20
Montbéliarde	61,881	522	63	186,368	0.81	0.18
Normande	78,472	526	45	190,280	0.82	0.17
Holstein	81,815	804	1059	201,554	0.81	0.17

^1^ Variants with a MAF ≥ 0.005 and with a Minimac imputation R² ≥ 0.20; ^2^ 2712 multi-breed sequences used for imputation (Table S1)

**Fig. 1:**
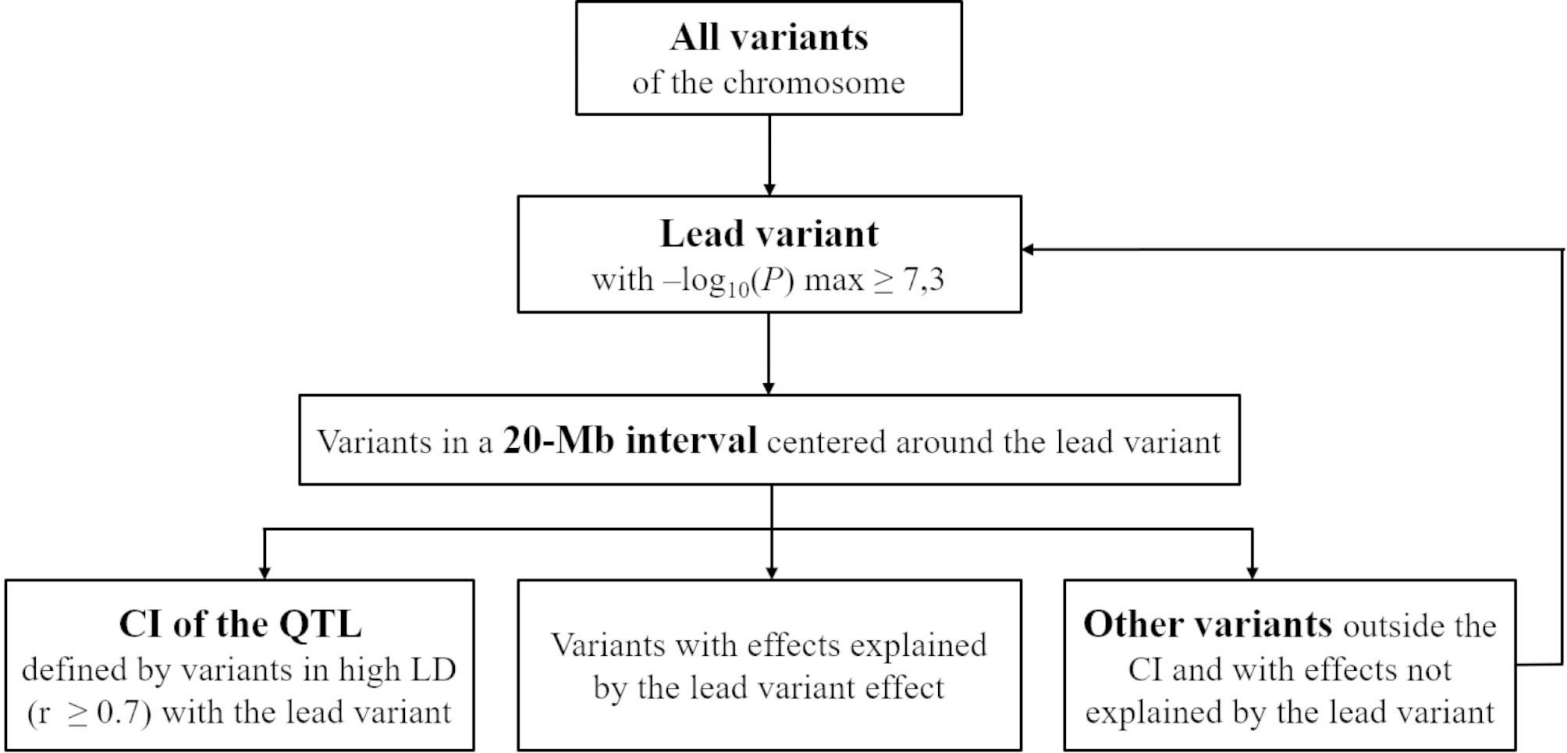
Iterative procedure for defining QTL and their confidence intervals

For each trait, we first conducted within-breed association analyses and then combined the within-breed results in a meta-analysis using the fixed effects method. The number of QTL and their confidence intervals were defined from both within-breed and meta-analysis results using an iterative procedure that evaluated linkage disequilibrium (LD) between the variant with the most significant effect, referred to as the lead variant, and variants located ± 10 Mbp around the lead variant (Fig. 1). The most plausible candidate genes were identified by considering the location of variants with the most significant effects in the QTL peaks.

Within-breed association analyses detected variants with significant effects (-log_10_(*P*) ≥ 7.3) in all breeds except Tarentaise and Vosgienne, and for all traits except HCR and CCR (Table 4, Table S2, Figs. S1 and S2). The number of QTLs identified in each breed varied widely, with 3 QTL detected in Abondance compared to 59, 71, and 72 QTL in Normande, Holstein, and Montbéliarde, respectively. The number of breed x trait combinations with significant effects also varied greatly depending on the trait analyzed: for example, 1 QTL was identified for MAST (in Abondance), while 19 were found for SCS (4, 4, and 11 in Montbéliarde, Normande and Holstein, respectively) and 35 for STAT (8, 13, and 14 in Montbéliarde, Normande and Holstein, respectively). Meta-analyses detected QTL for 8 of the 11 traits: 9 for PC, 15 for SCS and ICFI, 23 for PY, 24 for FY, 26 for STAT, and 27 for MY, but none for MAST, HCR, or CCR (Table 4, Table S3, Figs. S1 and S2).

**Table 4:** Number of QTL detected for the different traits in within-breed and meta-analyses of association

	MY	PY	FY	PC	FC	SCS	MAST	ICFI	STAT	Total
Abondance	0	0	1	1	0	0	1	0	0	3
Montbéliarde	14	11	6	13	11	4	0	7	8	74
Normande	12	8	4	9	6	4	0	3	13	59
Holstein	6	7	10	10	12	11	0	1	14	71
Meta-analyses	27	23	24	9	18	15	0	15	26	157

**Fig. 2:**
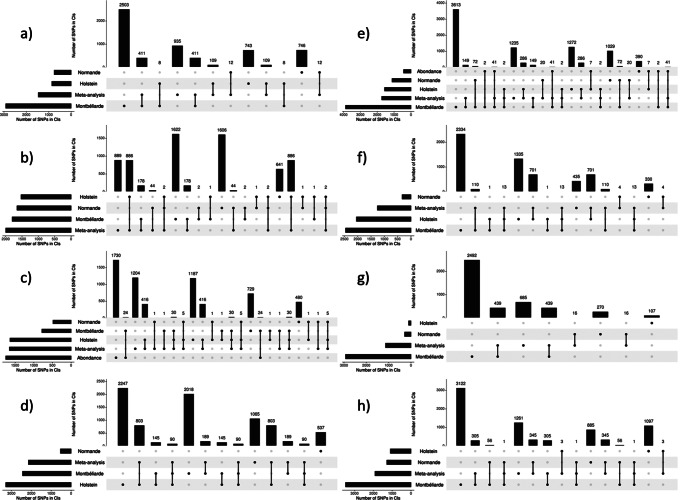
**UpSet diagrams for variants within the confidence intervals of QTL detected in within-breed and meta-analyses of association.** (a) milk yield (MY), (b) fat yield (FY), (c) protein yield (PY), (d) fat content (FC), (e) protein content (PC), (f) somatic cell score (SCS), (g) interval between calving and first insemination (ICFI), and (h) stature (STAT).

Features of all the QTL detected in within-breed analyses (Table S2) and in meta-analyses (Table S3) are summarized in Table 5. On average, the -log_10_(*P*) of the lead variant of a QTL was higher in Holstein, Montbéliarde, and the meta-analyses (13.8–14.2) than in Normande (11.2) and Abondance (8.6). Both in terms of the number of variants included and the length in Mbp, the average size of the confidence intervals of QTL was higher in Abondance (5.7 Mbp and 733 variants) and Montbéliarde (5.0 Mbp and 274 variants), lower in Holstein (2.8 Mbp and 151 variants) and Normande (2.5 Mbp and 96 variants), and lowest in the meta-analyses (1.7 Mbp and 65 variants). For each trait, Fig. 2 depicts the numbers of variants located within the confidence intervals of the QTL that were shared between the different within-breed analyses and the meta-analyses.

**Table 5:** Features of QTL detected for all traits in each within-breed and meta-analysis of association

			Confidence Interval	
		-log_10_(*P*) max	# variants	# size in kbp
	# QTL	Mean [min-max]	Mean [min-max]	Mean [min-max]
Abondance	3	8.6 [7.3–10.4]	733 [5–1754]	5775 [47–11,053]
Montbéliarde	74	13.8 [7.3–97.5]	274 [1–1411]	5064 [0–13,518]
Normande	59	11.2 [7.3–31.4]	96 [1–957]	2504 [0–13,190]
Holstein	71	14.2 [7.3–51.2]	151 [2–963]	2795 [7.7–13,425]
Meta-analyses	157	13.8 [7.3–111.9]	65 [1–570]	1743 [0–14,228]

Functional annotation of the lead variants for all QTL revealed that, both in within-breed and meta-analyses, the majority were intergenic (121 and 108, respectively) or intronic (61 and 34, respectively). Less frequently, lead variants were located in upstream regions (15 and 6, respectively), downstream regions (5 and 6, respectively), exons (missense: 2 and 1, respectively; synonymous: 2 in within-breed analyses), and 3’UTR regions (1 in within-breed analyses). The missense lead variants affecting *ENSBTAG00000006384* (FC in Montbéliarde), *NRK* (STAT in Normande), and *PLXNB3* (STAT in meta-analyses) genes were predicted to have SIFT moderate effects.

### Milk production and composition

**Fig. 3:**
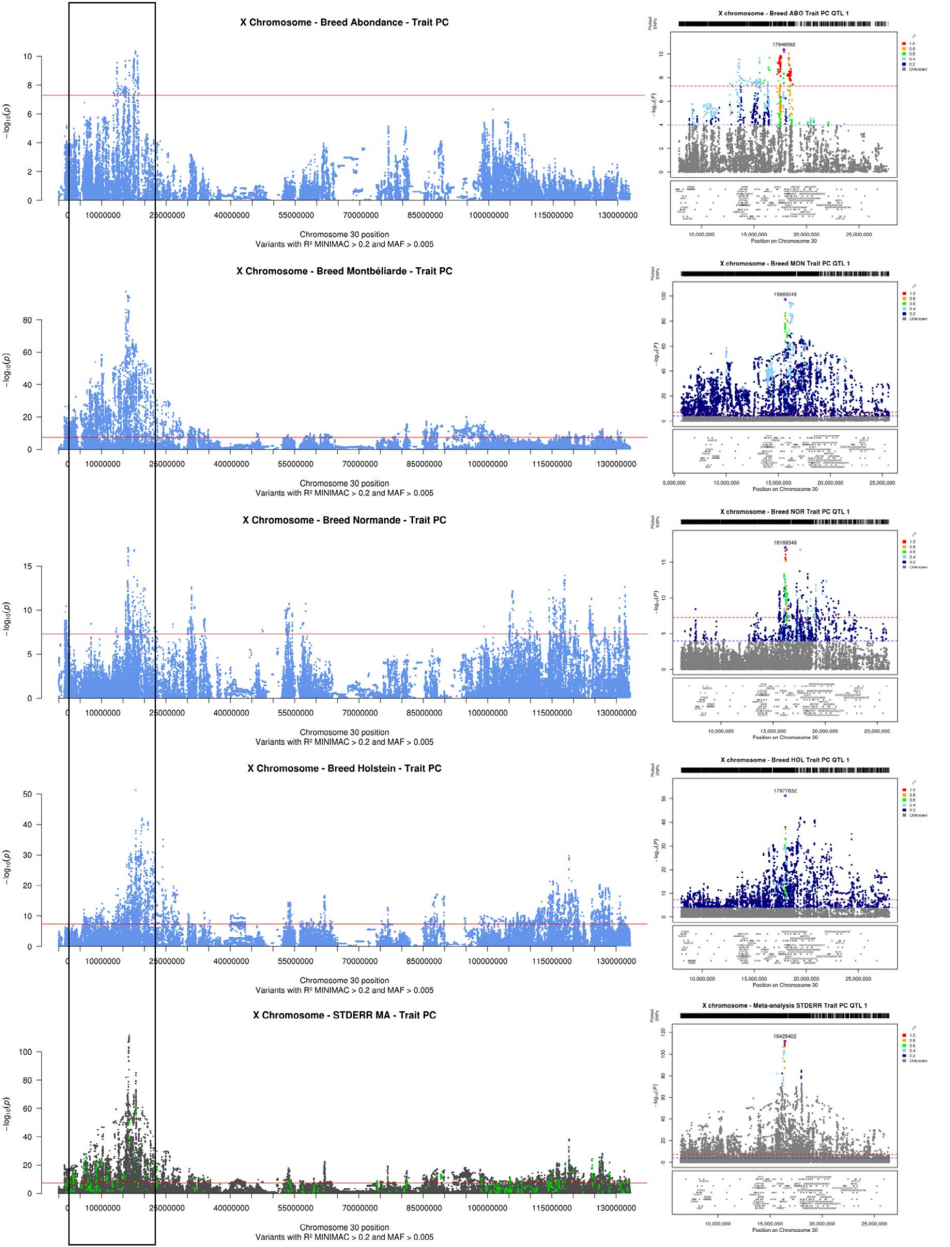
**Results of within-breed and meta-analyses of the X chromosome for protein content (PC): Manhattan plot for the entire chromosome and LocusZoom graph for the QTL with the most significant effects.** Within-breed association analyses in Abondance, Montbéliarde, Normande, and Holstein cows (Manhattan plot in blue); fixed effects meta-analyses (Manhattan plot in gray, variants with effects in the same direction in all within-breed analyses are highlighted in green); and corresponding LocusZoom graphs for the 20-Mb interval centered around the variant with the most significant effect

Across all of the within-breed association analyses, the QTL with the most significant effect was identified for PC in Montbéliarde, with the lead variant located at 15,665,045 bp (-log_10_(*P*) = 97.5). In the three other breeds for which QTL were detected, as well as in the meta-analyses, the QTL with the most significant effect was also found for PC, with the lead variant located at 17,846,562 bp in Abondance (-log_10_(*P*) = 10.4), 17,977,632 bp in Holstein (-log_10_(*P*) = 51.2), 16,169,349 bp in Normande (-log_10_(*P*) = 17.1), and 16,429,402 bp in meta-analyses (-log_10_(*P*) = 111.9) (Fig. 3). The confidence intervals of these QTLs were small in the meta-analysis (50 kbp with 19 variants), Holstein, and Normande (~ 200 kbp with 18 and 93 variants, respectively), but larger in Montbéliarde (1.9 Mbp with 73 variants) and Abondance (6.2 Mbp with 440 variants). Within the confidence intervals, the respective number of positional candidate genes was 2, 6, 4, 7, and 26. In all analyses but Holstein, the lead variant was located in an intergenic region; in Holstein, instead, it was located in the upstream region of the *MIR363* gene. In the vicinity of the lead intergenic variants, we identified the genes *GPC3*, *Metazoa_SRP*, *OR13H1*, *ENSBTAG00000051508, RAP2C, MBNL3*, and *HS6ST2* (Table S2). Across the different analyses, numerous other QTL were detected for PC (Fig. 2) and other milk production (MY, PY, and FY) and composition (FC) traits (Tables S2 and S3; Figs. S1 and S2). Similar to the QTL with the most significant effect on PC, several QTL detected for a given milk trait in different breeds were identified in neighboring regions, but only a few had their lead variants located in the same gene or close to the same gene in different breeds (Tables S2 and S3; Fig. 3). We note three in particular: (1) a QTL for FC detected in Normande and Montbéliarde with the lead variants located in an intronic region of *HS6ST2* (at 16,679,506 bp) and 250-kb upstream (at 16,420,927 bp), respectively; (2) a QTL for FY in both Normande and Holstein with lead variants located in introns of *DMD*, at 110,665,296 and 111,032,759 bp, respectively; and (3) a QTL for PY found in Holstein and Normande with the lead variants located in the same intergenic region between *DDX53* and *ENSBTAG00000049480*, at 120,448,964 and 120,590,087 bp, respectively. Interestingly, *HS6ST2*, *DMD*, and *DDX53* were also the most plausible positional candidate genes for QTL detected in the meta-analyses for FC, FY, and PY, respectively, but with different lead variants, located at 16,409,330 bp (intergenic), 110,665,443 bp (intronic), and 119,923,321 bp (intergenic), respectively.

### Udder health

**Fig. 4:**
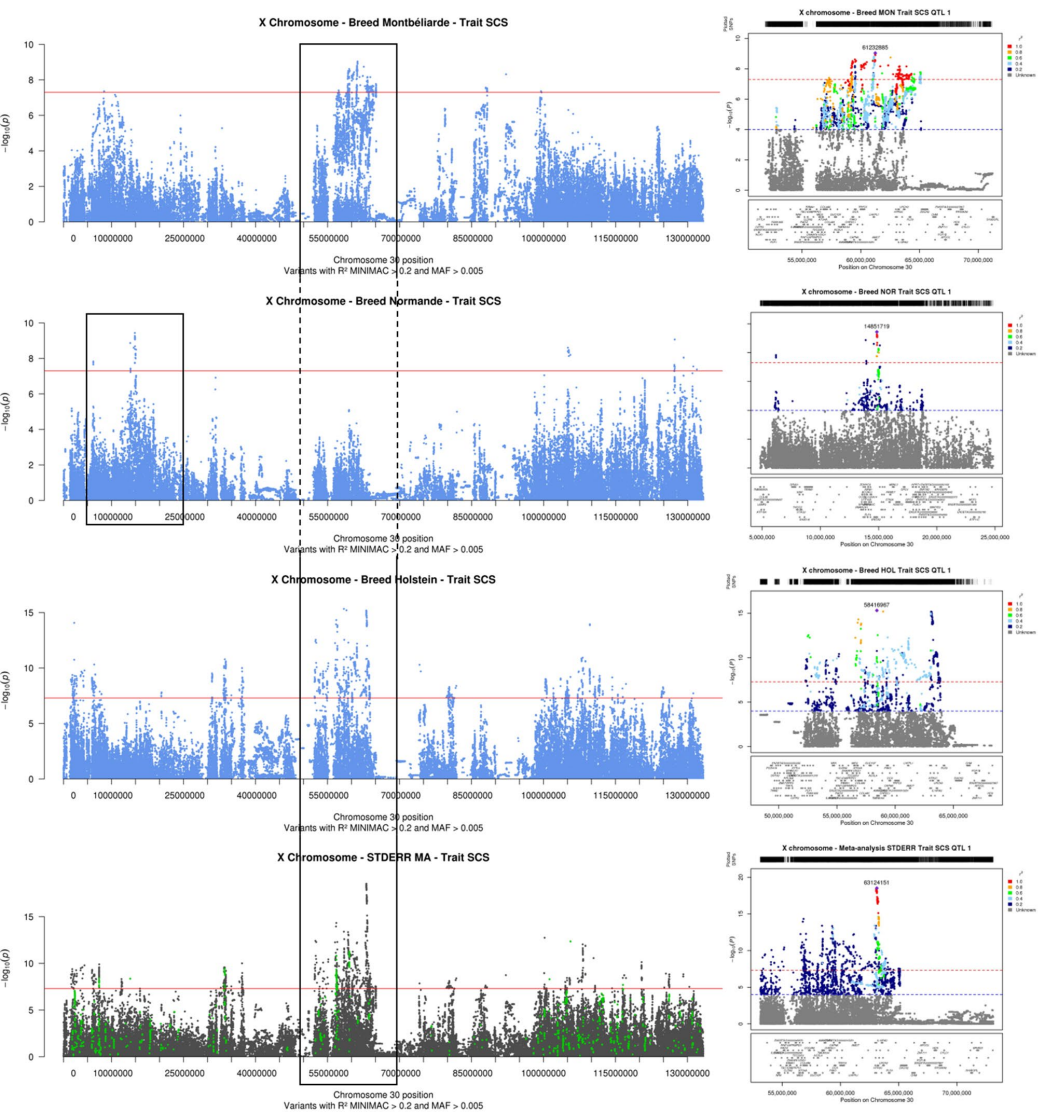
**Results of within-breed and meta-analyses of the X chromosome for somatic cell score (SCS): Manhattan plot for the entire chromosome and LocusZoom graph for the QTL with the most significant effects.** Within-breed association analyses in Montbéliarde, Normande, and Holstein cows (Manhattan plot in blue); fixed effects meta-analyses (Manhattan plot in gray, variants with effects in the same direction in all within-breed analyses are highlighted in green); and corresponding LocusZoom graphs for the 20-Mb interval centered around the variant with the most significant effect

While only one QTL was found for MAST (in Abondance), we detected 4, 4, and 11 QTL for SCS in Montbéliarde, Normande, and Holstein, respectively. These QTL were located in different regions in the different breeds, with *ACSL4*, *TMEM164*, *LHFPL1*, *AMOT*, and *ENOX2* as the most plausible positional candidate genes. Similarly, meta-analyses did not detect any QTL for MAST but did reveal 15 distinct QTL regions for SCS, with the one with the most significant effect located at 63.1 Mbp (intergenic lead variant between *bta-mir-1911* and *HTR2C*). In the vicinity of this region we also found the QTL with the most significant effects in Montbéliarde, with the lead variant located at 61,232,885 bp, 20 kbp downstream *AMOT*; however, the meta-analysis peak appeared much narrower, with a confidence interval of 671 kbp compared to 11.8 Mbp in Montbéliarde (Fig. 4). Other positional candidate genes that were identified in the meta-analyses for SCS included *ATG4A*, *TMEM47*, *ENSBTAG00000012533*, *TMEM187*, *IRAK1*, *PPP1R2C*, and *PPP4R3C*.

### Fertility

**Fig. 5:**
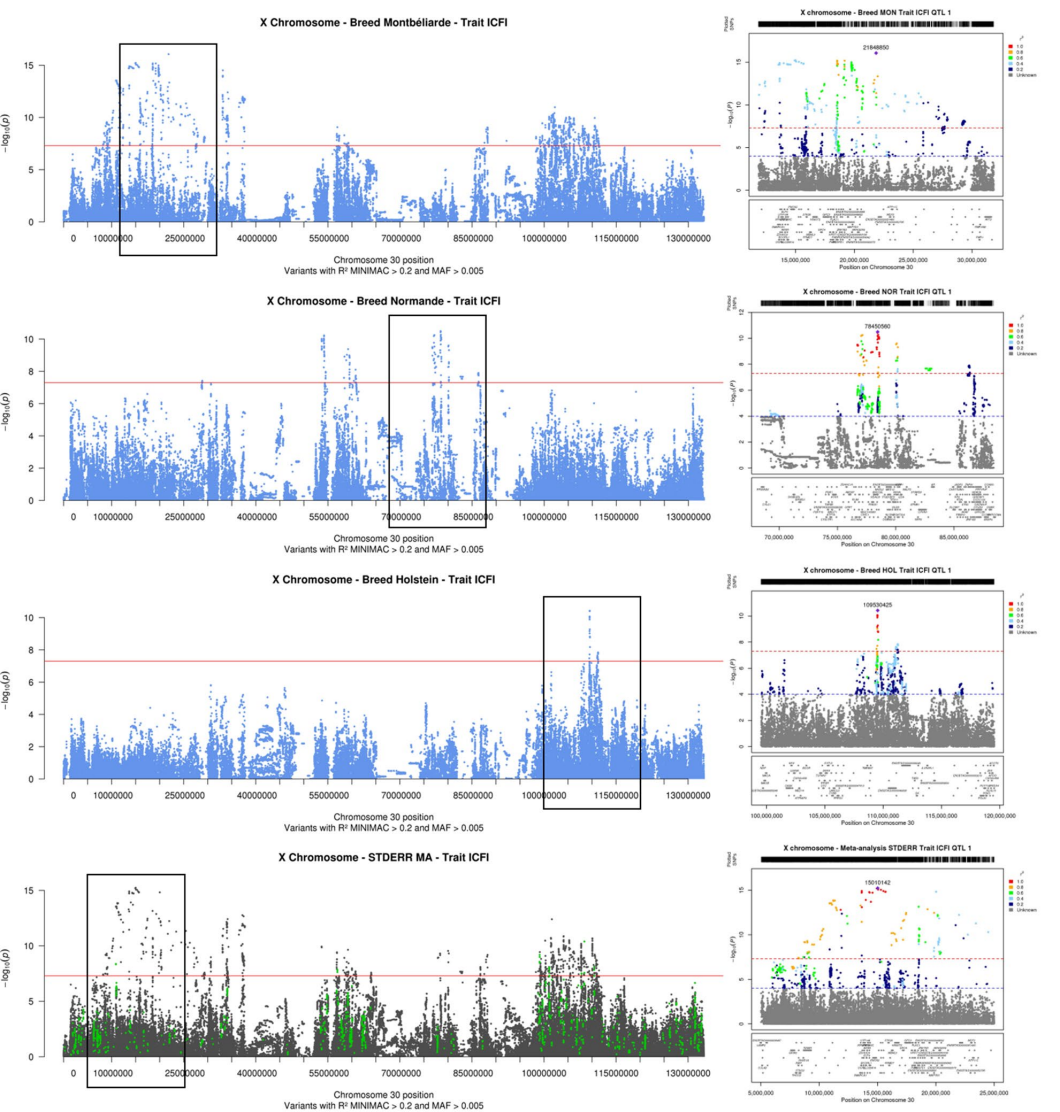
**Results of within-breed and meta-analyses of the X chromosome for interval between calving and first insemination (ICFI): Manhattan plot for the entire chromosome and LocusZoom graph for the QTL with the most significant effects.** Within-breed association analyses in Montbéliarde, Normande, and Holstein cows (Manhattan plot in blue); fixed effects meta-analyses (Manhattan plot in gray, variants with effects in the same direction in all within-breed analyses are highlighted in green); and corresponding LocusZoom graphs for the 20-Mb interval centered around the variant with the most significant effect

ICFI was the only fertility trait for which we detected QTL: 7 in Montbéliarde, 3 in Normande, 1 in Holstein, and 15 in the meta-analyses. As we observed in the SCS analyses, although the confidence intervals of some QTL overlapped between Montbéliarde and Normande, the lead variants were distinct. In addition, the QTL with the most significant effects were located in different regions in Montbéliarde, Normande, and Holstein (Fig. 5). In the within-breed analyses, the most significant QTL were highlighted in Montbéliarde: these included one intronic variant in *MAMLD1* and two intergenic variants between *ENSBTAG00000054511* and *PRR32* and between *ENSBTAG00000050383* and *ENSBTAG00000042114*, respectively. The meta-analysis revealed new QTL regions that were not found in within-breed analyses, with the most significant lead variant located between *ENOX2* and *ARHGAP36*.

### Stature

**Fig. 6:**
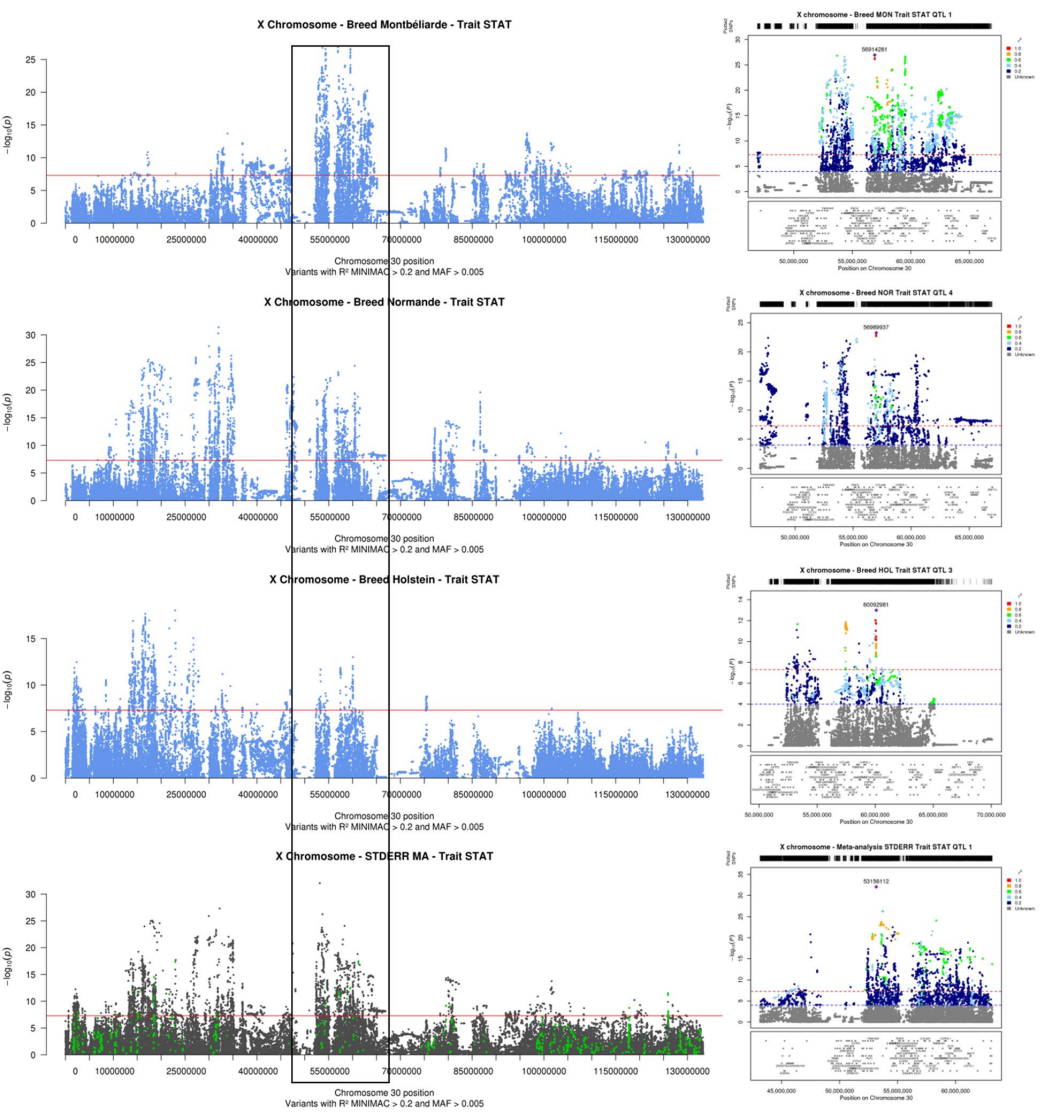
**Results of within-breed and meta-analyses of the X chromosome for stature (STAT): Manhattan plot for the entire chromosome and LocusZoom graph for the QTL with the most significant effects in meta-analyses.** Within-breed association analyses in Montbéliarde, Normande, and Holstein cows (Manhattan plot in blue); fixed effects meta-analyses (Manhattan plot in gray, variants with effects in the same direction in all within-breed analyses are highlighted in green); and corresponding LocusZoom graphs for the 20-Mb interval centered around the variant with the most significant effect in both the within-breed Montbéliarde analysis and meta-analysis

STAT was the trait for which the highest number of QTL was detected: 8 in Montbéliarde, 13 in Normande, 14 in Holstein, and 26 in the meta-anayses (Fig. 6). In Normande, Montbéliarde, and Holstein, the QTL with the most significant effects had their lead variant located in the downstream region of *IDS*, in an intron of *COL4A6*, and in the intergenic region between *ENSBTAG00000000567* and *ENSBTAG00000052795*, respectively. The *COL4A6* gene was also the most plausible positional candidate gene for another QTL detected in Normande, with a synonymous lead variant. Another gene, *TRPC5*, was highlighted in two breeds, but with two different intronic lead variants, at 60,006,622 bp in Normande and at 60,092,981 bp in Holstein. The most significant QTL detected in the meta-analysis had an intergenic lead variant located at 53,156,112 bp, between *ESX1* and *ENSBTAG00000053380*, and a large confidence interval (52.2–63.1 Mbp) that contained different QTLs than those found in the within-breed analyses, in particular those located near *COL4A6* and *TRPC5*. Certain candidate genes highlighted for STAT were also identified for other traits: two for PC (*MBLN3* and *GPC3*) and one for SCS (*PPP4R3C*). Finally, as mentioned above, the missense variants in *NRK* (g.54,703,522 C > T, p.714R > W) and *PLXNB3* (g.36994420G > A, p.1600 > H) genes were also noteworthy because for each of the QTL for which they were positional candidates, they are the variants with the most significant effect on STAT.

## Discussion

In mammals, the non-PAR region of the X chromosome differs from autosomes because it is hemizygous in males (XY). In females (XX), mechanisms of dosage compensation inactivate one copy of the X chromosome and thereby ensure the equal expression of these genes in both sexes. The complex mechanisms of X chromosome inactivation have been less extensively studied in cows than in other mammalian species but, in the absence of mutations affecting the Xist locus [25], both copies appear to be equally expressed in the mammary gland, which suggests random inactivation of either copy [17]. Because of these unique features, the X chromosome is usually excluded from genetic analyses. In cattle, previous efforts to test for genetic associations with the X chromosome examined reproductive traits in beef cattle at the 50k [26] and the HD [27–31] level. In the present study, instead, we focused on how the X chromosome affects the genetic determinism of 11 complex traits related to milk production and composition, udder health, fertility, and stature in six dairy cattle breeds. For each trait, we first estimated the proportion of genetic variance explained by this chromosome and then identified potential X-linked candidate genes and variants at the sequence level through within-breed and meta-analyses of association.

For the different traits and the different breeds, overall heritabilities—as estimated with genomic relationship matrices derived from EuroGMD autosomal and X-linked SNPs—were similar or higher to previous estimations obtained from pedigrees [32]. For each trait, the phenotypes used were yield deviations, i.e., mean performances adjusted for environmental effects. One potential issue with this approach is that, when records are repeated, the phenotype has a reduced residual variance and therefore a higher heritability. However, this phenomenon applies to all chromosomes equally and does not affect the proportion of genetic variance explained by the X chromosome.

Depending on the breed and trait in question, the gain in heritability due to inclusion of the X chromosome ranged from 0 to 0.04 points (+ 0.008 on average), indicating that the X chromosome can play an important role in the genetic determinism of traits of interest in dairy cattle. Generally speaking, values of h²_X_ were larger in the regional breeds with the lowest numbers of cows (Tarentaise and Vosgienne), and this was particularly true for the traits with the lowest heritabilities (MAST, CCR, HCR and ICFI). However, these results should be interpreted with caution due to the relatively large degrees of error associated with these estimates (e.g., h²_X_ = 0.011 ± 0.012 for ICFI in Vosgienne). Directly comparable results previously published are scarce for dairy cattle. In Holstein, VanRaden et al. [33] found the X chromosome accounted for about 1% of genetic variance of most of the 27 traits recorded in US and Canadian bulls while Su et al. [19] reported that using X chromosome SNPs resulted in a gain in the reliability of estimated breeding values averaged over 15 milk, fertility, udder health, and type traits (+ 0.3 to + 0.5% depending on the model used). Similarly, the heritability of female and male reproductive traits in beef cattle was estimated to increase by + 0.02 to + 0.09 as a result of the inclusion of X chromosome SNPs [34].

GWAS resolution is influenced by linkage disequilibrium. Overall, we show here higher levels of linkage disequilibrium on the X chromosome compared to an autosome of equivalent length (chromosome 2). Moreover, smaller breeds with reduced effective population size exhibited higher linkage disequilibrium. This distinction from autosomes is expected due to the greater genetic drift experienced by the X chromosome, as supporting by literature (e.g., [36]). Notably, the absence of segregation and recombination in male X chromosomes further impacts the mapping resolution of GWAS.

In our analyses, we considered paternal and maternal X chromosomes to be equally expressed at the population level and did not consider any potential effects of paternal versus maternal origin. In our association analyses, we applied the mixed model used for autosomes, which includes both the additive fixed effect of the variant tested and a vector of random polygenic effects to adjust the data for population structure. To capture the structure of the population and avoid detecting spurious associations, polygenic effects were estimated using a genomic relationship matrix calculated with the 50k SNPs of the autosomes only. We accounted for relatedness using a leave-one-chromosome-out (LOCO) approach, i.e., excluding the chromosome tested from the GRM. Although this approach may inflate test statistics [35], we chose to use it in order to avoid over-correction of the data and a consequent decrease in detection power, which was especially critical here because of the increased long-range LD on the X chromosome compared to autosomes [36]. The best option would have been to employ a leave-one-segment-out, or LOSO [37], approach that excluded the flanking region of the variant tested, but this was not computationally feasible due to the large number of animals and traits analyzed.

Regardless of the region of the genome studied, defining QTL regions (number and confidence intervals) from GWAS results is challenging and relies on a variety of more-or-less arbitrary approaches (e.g., LOD drop-off method [38]). In this study, we implemented a procedure that evaluated LD between the lead variant and variants in the surrounding region to (1) select variants to keep within the confidence interval and (2) search for other QTL on the chromosome after excluding the variants whose effects could be explained by that of the lead variant. This procedure, which can be compared to the COJO approach in GCTA software [39], has the advantage of being easily automated. Compared to autosomes, LD has a particularly strong influence in the non-PAR region of the X chromosome because no recombination occurs in males [36, 40]. Indeed, it is worth noting that the high number of QTL defined for some trait x breed combinations (up to 26 for STAT in meta-analyses), may be inflated due to the extreme LD in this region, which may have extended farther than the length of the window (20 Mbp) we used to define QTL. Increasing the size of the interval would have reduced the number of QTL but, because of the sequence-level resolution of our analyses, would also have caused computational issues. The number of QTL detected in this study is therefore probably an overestimate, but the procedure we developed would likely produce more accurate results in autosomes where the LD is less extensive.

We successfully identified QTL for most breeds and traits, with the exception of the two breeds with the lowest number of cows (Tarentaise and Vosgienne) and two fertility traits (HCR and CCR). As expected, due to the very different numbers of cows analyzed in each breed, the number of QTL was higher in the largest breeds, and these QTL presented more-significant effects. A higher number of QTL with more-significant effects was also found for the most heritable traits (milk production, milk composition, and stature).

Between breeds, we identified QTL with overlapping confidence intervals but the variants with the most significant effects were never the same, and were only rarely located in the same genes. This result has also been observed in autosomes (e.g., [41]), but appears to be more pronounced in the non-PAR region of the X chromosome, probably because of the heightened influence of within-breed LD. By combining different populations with different LD patterns, meta-analyses generally increase both the power and resolution of association analyses (e.g., [42]). Indeed, here this approach detected more breed x trait combinations with significant effects (n = 157), and, on average, more precise locations (65 variants in 1.7 Mbp), than any of the within-breed association analyses, for which the highest number of QTL detected was 74 (in Montbéliarde) and even the smallest confidence interval still contained 96 variants (in 2.5 Mbp in Normande).

As previously mentioned, the X chromosome has often been excluded from association analyses due to its unique pattern of inheritance, but it also suffers from a lower quality assembly and poorer functional annotation compared to autosomes, in particular in the previous UMD3.1 assembly [14, 17]. GWAS that examine the X chromosome are therefore rare, in particular at the sequence level. In the present study, by combining within-breed and meta-analyses of association at the sequence level on a large number of animals from six breeds and for a wide panel of traits, we are able to propose a list of candidate genes that could be responsible for the largest effects observed on the X chromosome.

Based on literature and human databases, the function of the positional candidate genes – or their association with traits similar to those investigated in this study – was examined in order to highlight the best functional candidates. Although we were not able to identify a clear functional link to milk production and composition traits, four of the best positional candidate genes associated with the most significant QTL for these traits warrant special attention: *GPC3* (*glypican 3*), *MBNL3* (*muscleblind like splicing regulator3*), *HS6ST2* (*heparan sulfate 6-o sulfotransferase 2*), and *DMD* (*dystrophin*). *GPC3*, which was one of the best candidates for PC in our study, has been previously associated to longevity [43] and metabolic disorders [44] in Holstein cows; both traits could be related to milk composition. A more recent study, conducted at the whole-genome sequence level, identified a QTL for milk urea nitrogen (which is genetically correlated with milk PC [45]), in which the lead SNP was located between *MBNL3* and *HS6ST2* at 16,376,624 bp [46], i.e., close to the lead variants we found for several QTL for PC in Normande (at 16,169,349 bp), and FC in Montbéliarde (at 16,420,927 bp) and Normande (at 16,679,506 bp). Furthermore, *HS6ST2* knockout mice showed glucose and insulin metabolism disorders [47]. Mutations in the *DMD* gene, leading to the absence or dysfunction of the dystrophin protein causing muscular dystrophies in humans, were associated with muscle fat replacement [48]. In a Chinese Holstein population, genome-wide association analyses of milk, protein, and fat yields, conducted with SNPs of the Illumina BovineSNP150 BeadChip, identified rs135780687, located at 127,465,011 bp on the X chromosome, as the lead SNP for a QTL detected for FY [49]. The authors identified *GRPR*, encoding a gastrin-releasing peptide receptor, as a functional candidate gene in this region. In the present, sequence-level, study, we detected QTL in this region for multiple traits but the most plausible positional candidate genes were generally different from *GRPR*, with the exception of two QTL identified in meta-analyses for MY and PY, for which the lead variants were located in the intergenic region between the *GRPR* and *AP1S2* genes (at 127,509,209 and 127,507,371 bp, respectively).

Notably, among the positional candidate genes identified for udder health, three encode transmembrane proteins (*TMEM164*, *TMEM47*, and *TMEM187*) and two encode protein phosphatase regulators (*PPP1R2C* and *PPP4R3C*). The lead variant of the QTL with the most significant effect on SCS in Holstein was located close to *TMEM164*, which was found to be differentially expressed in mammary infections due to *E. coli* and *S. aureus* in cattle [50] and associated with improved survival and increased immune cell infiltration in patients with pancreatic cancer [51]. *ACSL4* (*acyl-CoA synthetase long chain family member4*), which is involved in fatty acid metabolism, is another positional candidate gene for the most significant QTL identified in Holstein. This gene was reported to be upregulated in late lactation, resulting in an increased concentration of triglycerides in bovine mammary epithelial cells [52]; interestingly, Genini et al. [50] demonstrated that lipid metabolism was significantly affected during the cattle-specific response to mastitis infection, suggesting that it could be tightly linked to immune response. Furthermore, *ACSL4* was found to be involved in inflammatory responses in mice [53]. Two other genes, candidates for the most significant QTL identified in Normande (*ENOX2*, *ecto-NOX disulfide-thiol exchanger 2*) and in meta-analyses (*HTR2C*, *5-hydroxytryptamine receptor 2 C*), have been associated with ruminant health: the former was associated with innate immunity in sheep [54] while the latter was identified as a candidate gene for hyperketonemia in Holstein cows [55]. Finally, two other genes are noteworthy: *AMOT* (*angiomotin*), associated with the most significant effects on SCS in Montbéliarde, promotes the proliferation of mammary epithelial cells in women [56] and *IRAK1* (interleukin 1 receptor associated kinase 1), one of the best candidates in both Holstein and meta-analyses, was described as a critical signaling mediator of innate immunity [57].

Among the genes identified for ICFI, which was the only fertility trait with significant results in our study, *MAMLD1* and *COL4A6* appear to be the best functional candidates. *MAMLD1* (*mastermind-like domain containing 1*) has been associated with disorders of sex development in men, as well with ovarian dysfunction in women [58, 59]. This gene has also been highlighted as a candidate for bull fertility [27] and for the number of piglets born alive [60]. *COL4A6* (*collagen type IV alpha 6 chain*) was found to be particularly expressed in the bovine uterus [61] and differentially expressed in the endometrium of high- and low-fertility heifers during the mid-luteal phase of the estrus cycle [62].

Because of its relatively high heritability and ease of measurement, stature (height in humans) has been examined in very large-scale GWAS and meta-analyses in different species, including cattle [10] and humans [3]. However, neither one of these studies considered the X chromosome, which could explain a part of the missing heritability observed in each case. Indeed, in the present study, stature was the trait for which the highest number of QTL was detected (8 in Montbéliarde, 13 in Normande, 14 in Holstein, and 26 in meta-analyses) and we identified a number of functional candidate genes that might explain the effects we observed. For the QTL located around 53 Mbp, the confidence intervals are probably inflated due by the long order inversion between 51.9 and 54.5 Mbp on the ARS-UCD1.2 bovine assembly previously identified by Zhang et al. [40]. In particular, although they were predicted to have only moderate effects, two of the lead variants were associated with missense changes in the proteins encoded by *NRK* (*nik-related protein kinase*), at 54,703,522 bp, and *PLXNB3* (*plexin B3*), at 36,994,420 bp. Interestingly, *NRK* has been previously linked with an X-linked form of short stature in humans [63], which supports its role in the genetic determinism of this trait. In contrast, no functional link with stature has yet been identified for *PLXNB3*. Furthermore, a larger number of positional candidate genes for stature were shared between breeds than any of the other traits we examined. Among these genes, the best positional candidates were *TRPC5* (*transient receptor potential cation channel subfamily C member 5*), which was shared between Holstein and Normande, and *COL4A6* (*collagen type IV alpha 6 chain*), which was detected in both Montbéliarde and Normande. The latter gene was also associated with fertility in our study, but presented no obvious functional link with stature. Among the within-breed analyses and the meta-analysis, several other genes were highlighted for the QTL with the most significant effects, namely, *GPR50* (*G protein-coupled receptor 50*), *VMA21* (*vacuolar ATPase assembly factor VMA21*), *IDS* (*iduronate 2-sulfatase*), *GPC4* (*glypican 4*), *ESX1* (*ESX homeobox 1*), *GPC3* (*glypican 3*), *bta-mir-507b*, *PCDH19* (*protocadherin 19*), and *SLITRK4* (*SLIT and NTRK like family member 4*). Four of these are involved in growth disorders in humans: *ESX1* and *GPR50* were found to be involved in growth and pituitary hormone deficiencies [64, 65], while *GPC3* and *GPC4* were identified as genes causing Simpson-Golabi-Behmel syndrome, a rare X-linked syndrome characterized by pre‐and post‐natal overgrowth [66].

## Conclusions

With its large sample size and fine-scale (sequence-level) resolution, this study provides clear evidence for the importance of the X chromosome in the genetic determinism of complex traits in dairy cattle. These new insights support the inclusion of this chromosome in all genetic evaluation models in which it is not currently considered. Based on our results, we would expect that inclusion of the X chromosome would increase the accuracy of estimated breeding values and expedite genetic progress on milk production, milk composition, udder health, and fertility traits, which are all included in the breeding goals of the different dairy cattle breeds. Although the necessary changes to the evaluation software and procedure would not be trivial, the relative gain of 3–5% in the accuracy of genomic estimated breeding values would justify the effort. In addition, because the majority of X-linked genes are shared between mammals, further work on the identification of X-linked genes that are involved in the genetic determinism of traits could be beneficial for our understanding of other species.

## Methods

### Ethics statement

All analyses were performed using data from routine milk recording and genotyping in commercial herds of French cows. We did not perform any experiments on animals and no ethical approval was required.

### Cows, phenotypes, and genotypes

We analyzed 236,496 cows from three national breeds—Holstein (81,815 animals), Montbéliarde (61,881 animals), and Normande (78,472 animals)—and three regional breeds—Abondance (7449 animals), Tarentaise (3969 animals), and Vosgienne (2910 animals)—for which phenotypes and 50k genotypes were available (Table 1).

Phenotypes were obtained for 11 traits:

- Five milk production traits defined at the level of the individual lactation based on monthly records: milk yield (MY), protein yield (PY), fat yield (FY), protein content (PC), and fat content (FC);
- Two udder health traits: average somatic cell score (SCS) at lactation level, computed as the mean of monthly records of log-transformed somatic cell counts, and clinical mastitis (MAST; except for Vosgienne) defined as 0/1 (0 = no clinical mastitis and 1 = at least one episode of clinical mastitis in the interval from 10 days before calving to 150 days after calving);
- Three female reproductive traits: the interval between calving and the first artificial insemination (ICFI) which reflects the ability of a cow to initiate the postpartum cycle, and heifers’ (HCR) and lactating cows’ (CCR) conception rates, which represent the success/failure (1/0) of each artificial insemination;
- Stature (STAT);
- To remove the influence of environmental effects, all phenotypes used were yield deviations (YD), i.e., phenotypes adjusted for non-genetic effects and, for repeated records, averaged per cow. YDs are produced by the French national genetic evaluation systems for the Holstein, Montbéliarde, Normande, Abondance, Tarentaise, and Vosgienne populations using the models described at https://interbull.org/ib/geforms [67].

Cows in the six breeds were genotyped with different versions of the 50 K SNP Beadchip, with the most recent being the EuroGMD Beadchip, which is currently used for genomic selection (https://www.eurogenomics.com/actualites/the-eurog-md:-a-unique-genotyping-microarray-for-cattle-.html). The standard EuroGMD Beadchip contains 53,469 autosomal SNPs and 1147 SNPs located in the non-pseudoautosomal region (non-PAR) of the X chromosome; all SNPs passed all quality control filters (individual call rate > 95%; SNP call rate > 90%; minor allele frequency (MAF) > 1%; genotype frequencies in HW equilibrium with P > 10^− 4^).

### Imputation analyses

Missing genotypes of EuroGMD SNPs are routinely imputed in the French evaluation system using FImpute software [68]. For imputations at higher densities (HD and sequence levels), we considered only the X-specific non-PAR region, which covers the majority of the X chromosome (0–133.3 Mbp on the ARS-UCD1.2 reference genome [14]). To account for male hemizygosity for this chromosome, we assumed that all males were homozygous for all non-PAR SNPs, and removed the pedigree information. All imputation analyses were done within-breed. First, HD genotypes of 32,268 SNPs were imputed with FImpute [68] from genotypes of the 1147 non-PAR SNPs of the EuroGMD chip, using the 179 to 804 major ancestors of each breed with HD genotypes as a reference (Table 3). Then, 778,576 sequence variants were imputed using a multi-breed population of 2712 animals from the RUN8 reference panel of the 1000 Bull Genomes consortium [10] and the Minimac algorithm [24]. The reference population for the sequence-level imputation comprised 2712 *Bos taurus* animals from 28 different breeds, including 1059 Holstein, 63 Montbéliarde, 45 Normande, 9 Abondance, 12 Tarentaise, and 4 Vosgienne (S1 Table).

### Linkage disequilibrium

Linkage disequilibrium (r2) were assessed for the X chromosome and for an autosome of equivalent length (chromosome 2) in each breed using a sample of cows. To minimize relatedness and avoid accumulation of the same paternal X chromosome, one daughter per sire was randomly selected. A total of 126 to 2279 individuals were included in the study, depending on the breed. High density SNP genotypes, either true or imputed, were used for a total of 15,892 and 35,723 SNPs on chromosomes X and 2, respectively. Variants with a minor allele frequency (MAF) less than 0.01 were excluded from the analysis. The calculated values were then averaged within bins based on marker distance.

### Genomic relationship matrices

Three different genomic relationship matrices were constructed at the 50k density: the first contained 53,469 autosomal SNPs (**G**_**A**_), the second contained 1147 SNPs of the non-PAR X chromosome (**G**_**X**_), and the third contained both autosomal and X-chromosome SNPs, i.e., 54,616 SNPs (**G**_**G**_). As all animals included in this study were females, no assumptions were made regarding dosage compensation for the X chromosome (both X chromosomes active in females). All matrices were therefore constructed using the --make-grm option of GCTA software [69] which was developed for autosomal SNPs.

### REML analyses

To estimate the relative proportions of genetic variance explained by the autosomes and the X chromosome, and the corresponding heritabilities, within-breed REML analyses were carried out for each trait using GCTA software [69] and the following model:

1 $$y = 1\mu + {g_A} + {g_X} + e,$$

where **y** is the vector of YD; µ is the overall mean; **g**_**A**_**~** N(0,**G**_**A**_σ²_A_) is the vector of random autosomal genetic effects, with **G**_**A**_ the autosomal GRM and σ²_A_ the autosomal genetic variance; **g**_**X**_**~** N(0,**G**_**X**_σ²_X_) is the vector of random X-linked genetic effects, with **G**_**X**_ the X-chromosome GRM and σ²_X_ the X-linked genetic variance; and **e ~** N(**0**,**I**σ²_e_) is the vector of random residual effects, with **I** the identity matrix and σ²_e_ the residual variance.

Then, we calculated the overall heritability of the traits (**h²**=(σ²_A_+σ²_X_)/(σ²_A_+σ²_X_+σ²_e_)) and the heritability due to autosomes (**h²**_**AUT**_=σ²_A_/(σ²_A_+σ²_X_+σ²_e_)) and the X chromosome (**h²**_**X**_=σ²_X_/(σ²_A_+σ²_X_+σ²_e_)).

### Within-breed association analyses

Allele dosages of X-linked variants imputed at the sequence level were evaluated in within-breed association analyses using GCTA software [69]. To adjust data for population structure, we estimated polygenic effects using the **G**_**A**_ GRM calculated with autosomal 50k SNPs. All phenotypes were measured on females, which had two copies of the X chromosome. Therefore, we applied the following linear mixed model:

2 $$y = 1\mu + xb + {g_A} + e,$$

where **y** is the vector of YD; µ is the overall mean; b is the additive fixed effect of the variant tested; **x** is the vector of imputed allele dosages; **g**_**A**_**~** N(0, **G**_**A**_ σ²_A_) is the vector of random polygenic effects, with **G**_**A**_ the GRM based on autosomal SNPs and σ²_A_ the autosomal polygenic variance; and **e ~** N(**0**,**I**σ²_e_) is the vector of random residual effects, with **I** the identity matrix and σ²_e_ the residual variance.

We analyzed variants with a MAF ≥ 0.005 and with a Minimac imputation R² ≥ 0.20, which resulted in between 154,966 and 201,554 variants depending on the breed (Table 3).

### Association meta-analyses

We then conducted meta-analyses for each trait by combining the within-breed association results of the six breeds (five for MAST, which was not measured in Vosgienne). All variants retained after filtering (MAF ≥ 0.005 and Minimac R² ≥ 0.20), i.e., 212,111 for MAST and 224,073 for all other traits, were included in meta-analyses. The fixed effects meta-analysis method was applied as implemented in METAL software [70]. This method assumes that the true effect of each allele is the same across different studies and combines the different effects by weighting them by the inverse of their error variance. Therefore, this meta-analysis method weights the different studies by their sample size.

### Identification of QTL regions

We implemented an iterative procedure to identify QTL regions in the within-breed association analyses and meta-analyses based on a threshold corresponding to P = 0.05 after Bonferroni correction for ~ 1 million independent tests (-log_10_(*P*) = 7.3) [71].

For each trait, we applied the following six-step iterative procedure:

1. Search for the variant with the maximal -log_10_(*P*) (≥ 7.3), hereafter named the lead variant;
2. Select all variants within a 20-Mb window centered around the lead variant;
3. Calculate LD between the lead variant and all other variants in the 20-Mb interval, i.e., correlations between allele dosages (r);
4. Define the confidence interval of the QTL by retaining all variants in high LD with the lead variant (|r| ≥ 0.7);
5. For each variant *i* outside of the confidence interval and located in the 20-Mb window, determine if its effect could be explained by the effect of the lead variant; for this, we calculated a new test statistic, T_NEW_ = (b_i_ – b_LEAD_ x r (i,LEAD)) / SE_i_, and the corresponding log_10_(*P*_NEW_) value, and selected variants that satisfied the following two conditions: (1) log_10_(*P*_NEW_) < 7.3 and (2) T_i_ (b_i_/SE_i_) and T_NEW_ had the same sign;
6. Remove all variants identified in steps 4 (variants within the confidence interval) and 5 (variants with effects explained by LD with the lead variant).

This procedure, described in Fig. 1, was repeated until no more significant variants were found on the chromosome.

**Functional annotation and visualization**.

Variants in the confidence intervals of each QTL were annotated with the Ensembl variant effect predictor (VEP) pipeline v81 [72] and effects of amino-acid changes were predicted using the SIFT tool [73]. Functions of genes were investigated using GeneCards [64] and MalaCards [74]. Visualization of the QTL and of their annotation was performed using the R LocusZoom function [75]. Manhattan plot and UpSet diagrams were created using the R packages qqman [76] and UpSetR [77], respectively.

## Electronic supplementary material

Below is the link to the electronic supplementary material.

Supplementary Material 1

Supplementary Material 2

Supplementary Material 3

Supplementary Material 4

Supplementary Material 5

## Data Availability

All GWAS and meta-analyses results are available at the following URL https://entrepot.recherche.data.gouv.fr/privateurl.xhtml?token=fb349842-2a69-49d5-bdca-a3f0ec3d1dbd. Raw genotypes and phenotypes data are part of a reference population used for genomic selection and have commercial value. Therefore, restrictions apply to their availability and they are not publicly available. The authors can be contacted for a reasonable request.

## Declarations

**Ethics approval and consent to participate**.

**Consent for publication**.

## List of Abbreviations

CCR: Lactating cows’ conception rate
FC: Fat content
FY: Fat yield
GRM: Genomic relationship matrix
GWAS: Genome-wide association studies
HCR: Heifers’ conception rate
HD: High density
ICFI: Interval between calving and first fertilizing artificial insemination
LD: Linkage disequilibrium
LOCO: Leave-one-chromosome-out
LOSO: Leave-one-segment-out
MAF: Minor allele frequency
MAST: Clinical mastitis
MY: Milk yield
non-PAR: Non-pseudoautosomal region
PC: Protein content
PY: Protein yield
QTL: Quantitative trait locus
REML: Restricted maximum likelihood
SCS: Somatic cell score
SNP: Single nucleotide polymorphism
STAT: Stature
