# Supplementary figures and images for: X-linked genes influence various complex traits in dairy cattle
Source: BMC Genomics. 2023 Jun 19;24:338. doi: 10.1186/s12864-023-09438-7 (PMC10278306; doi:10.1186/s12864-023-09438-7)

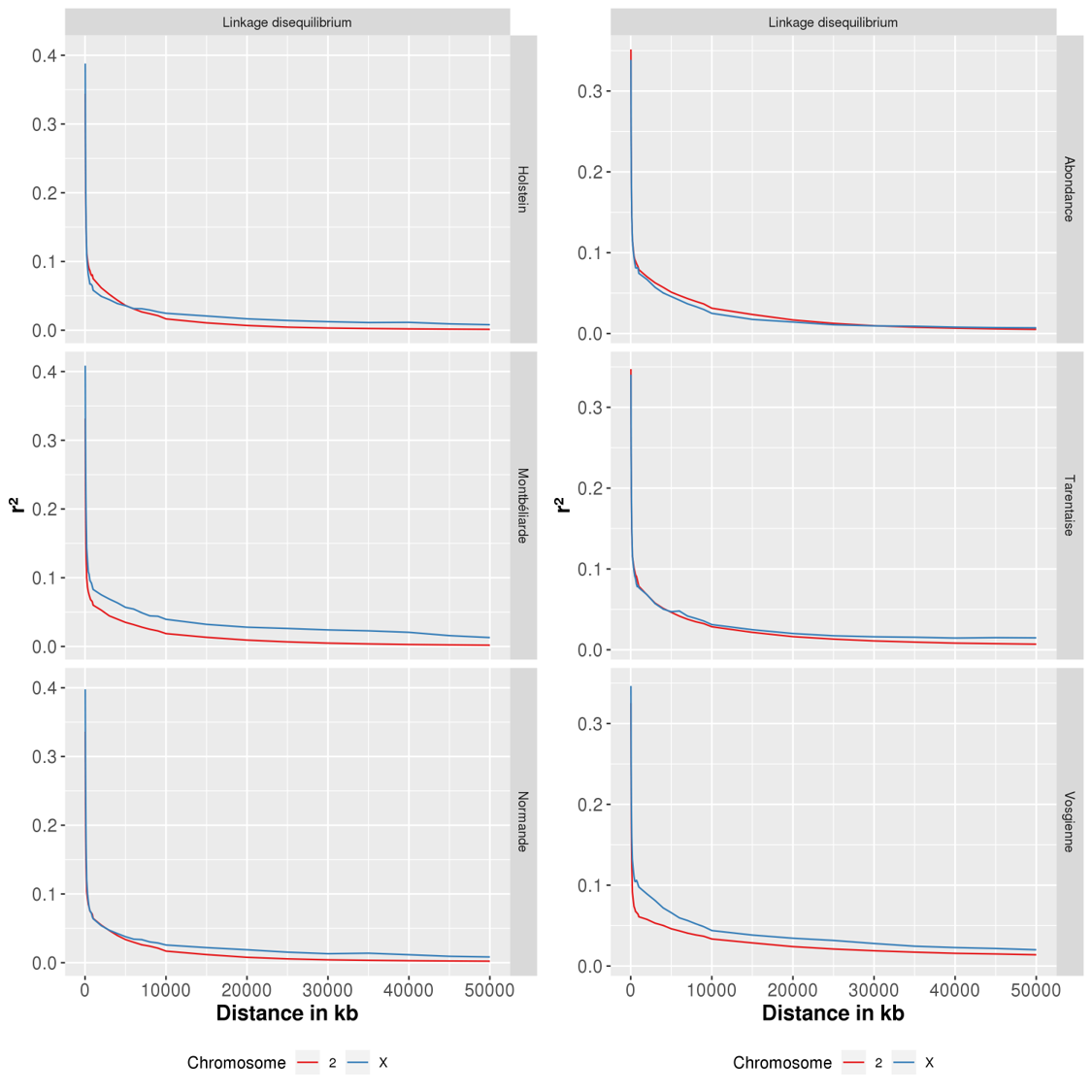

Supplement: Supplementary file 1 — Supplementary Material 1 [file 12864_2023_9438_MOESM1_ESM.png]

a)

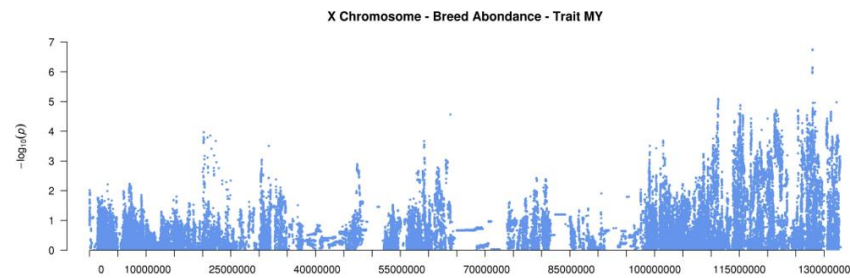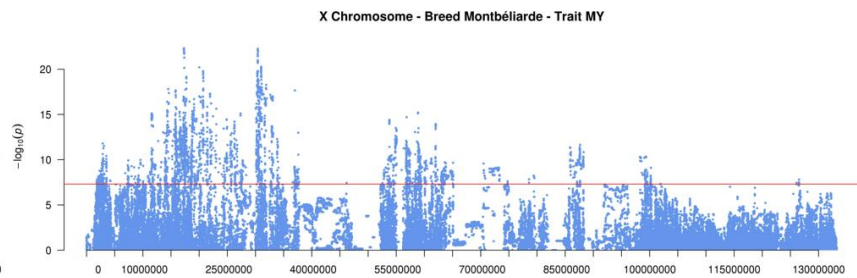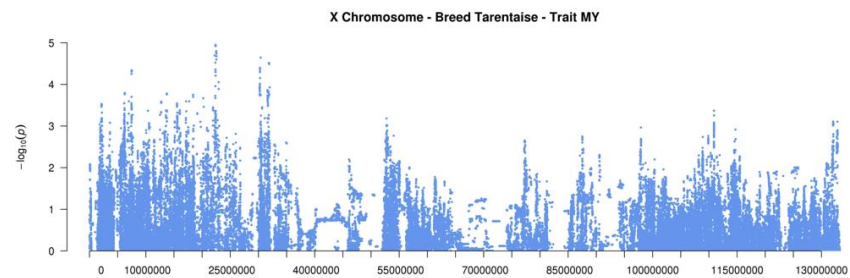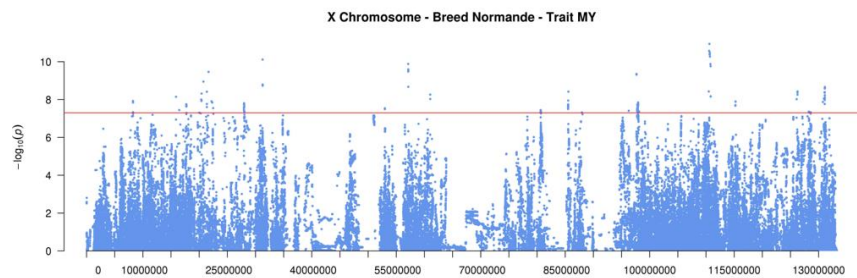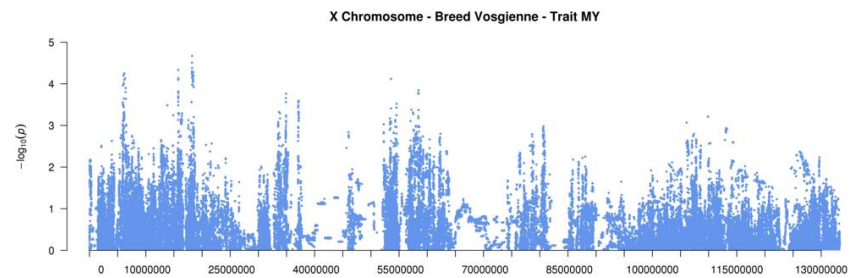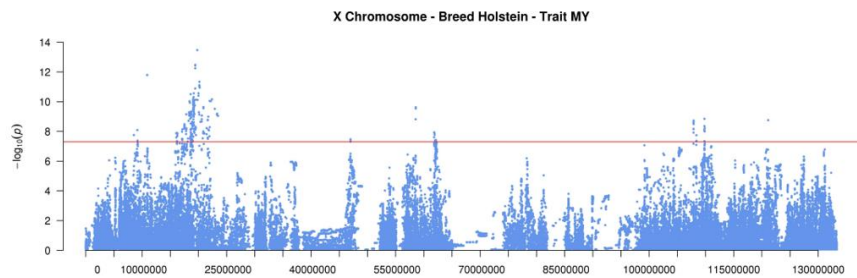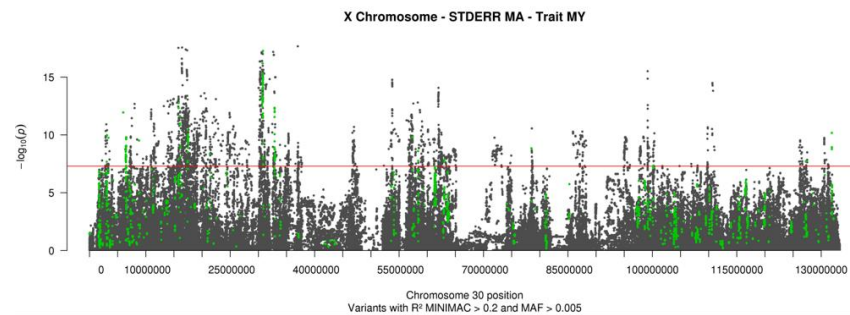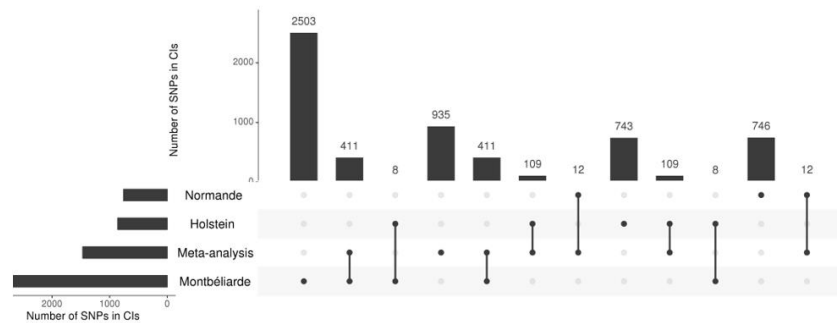

**b)**

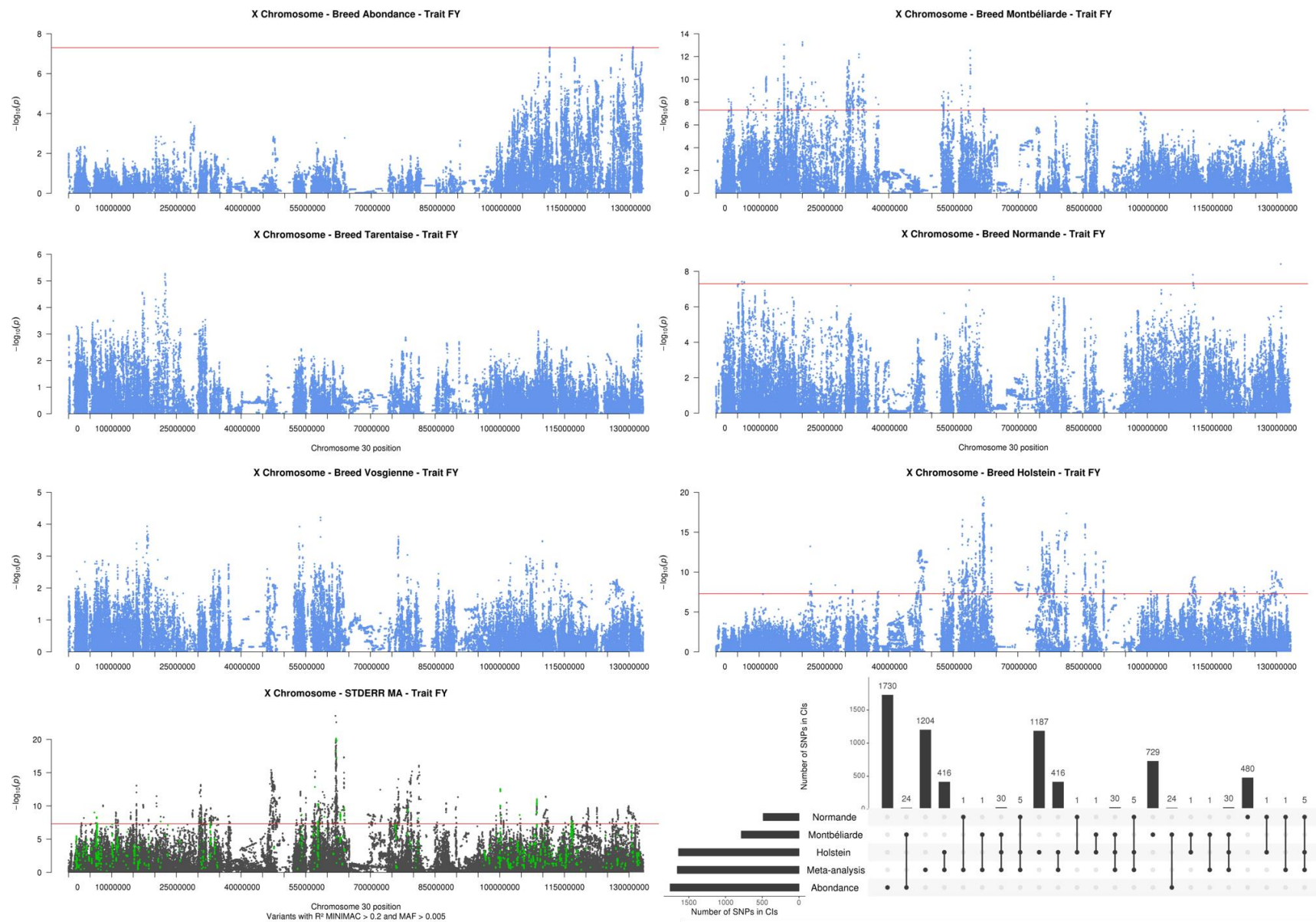

c)

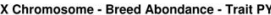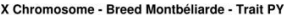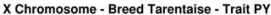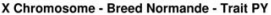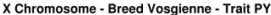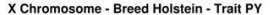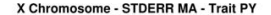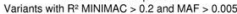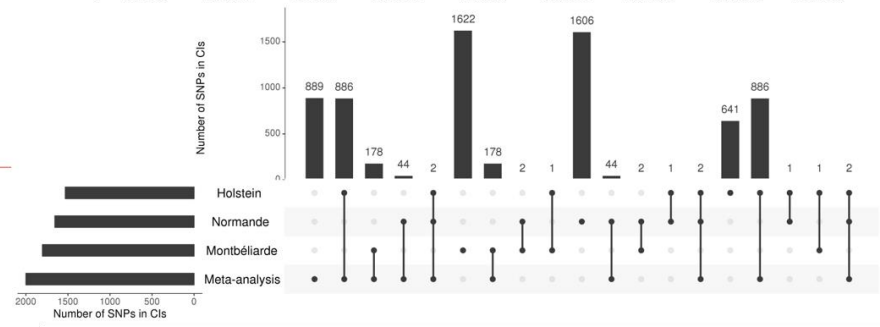

d)

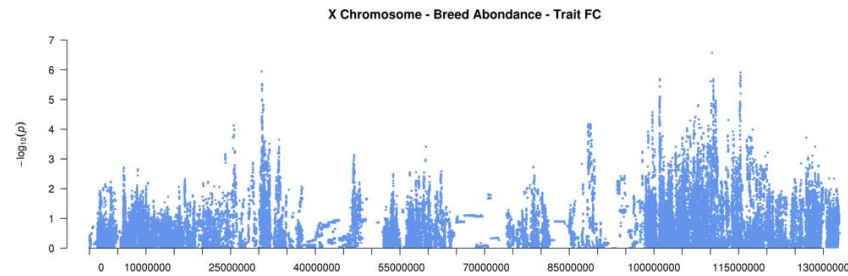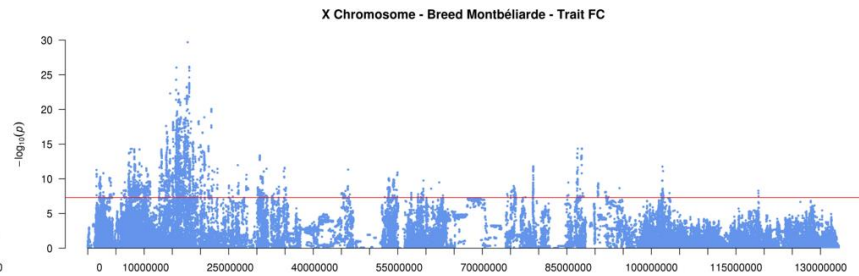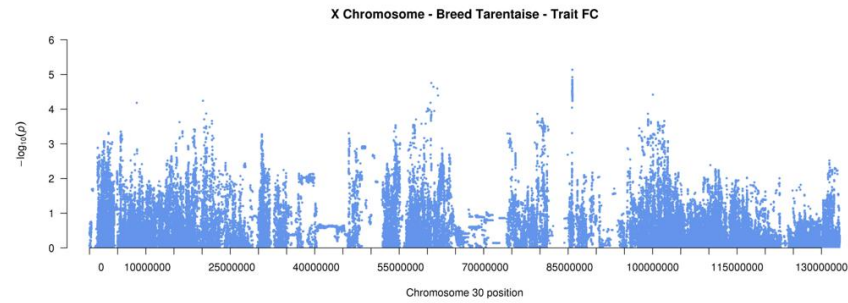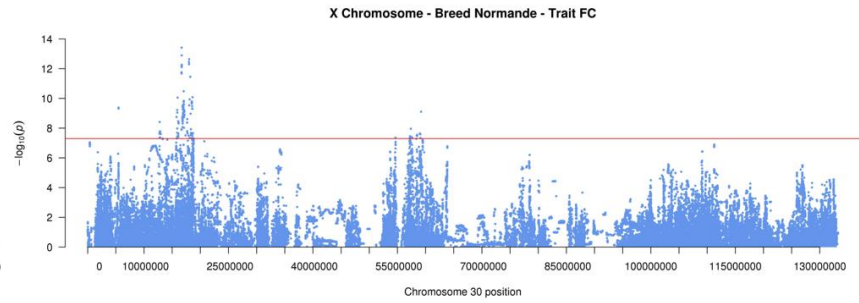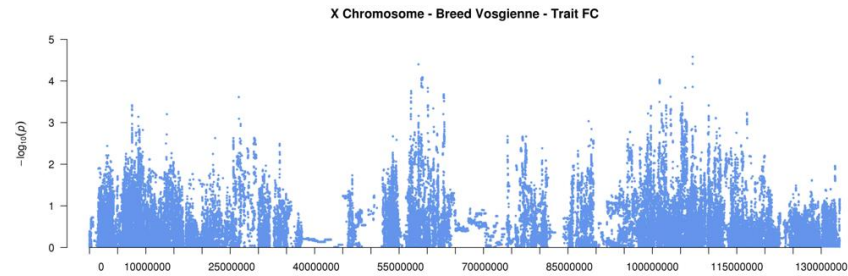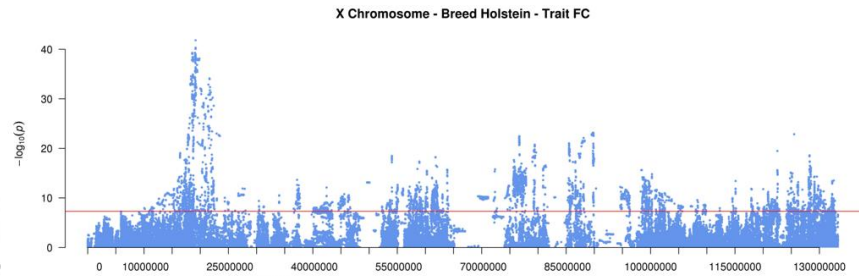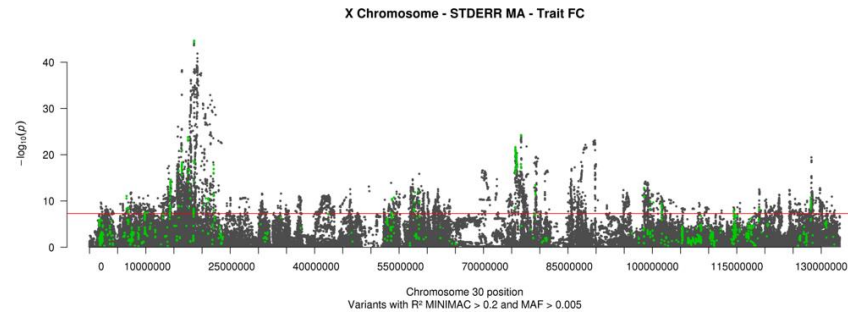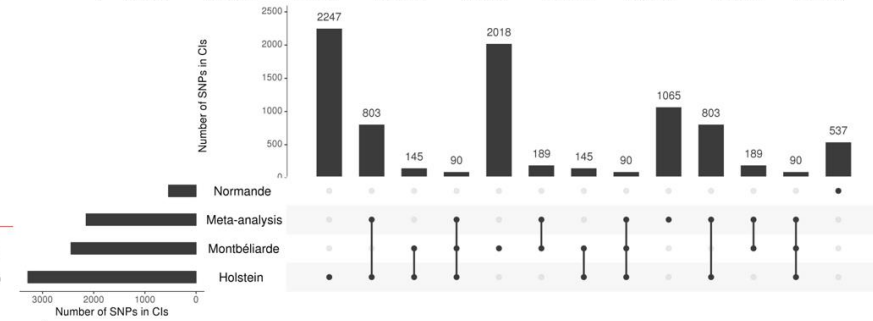



**f)**

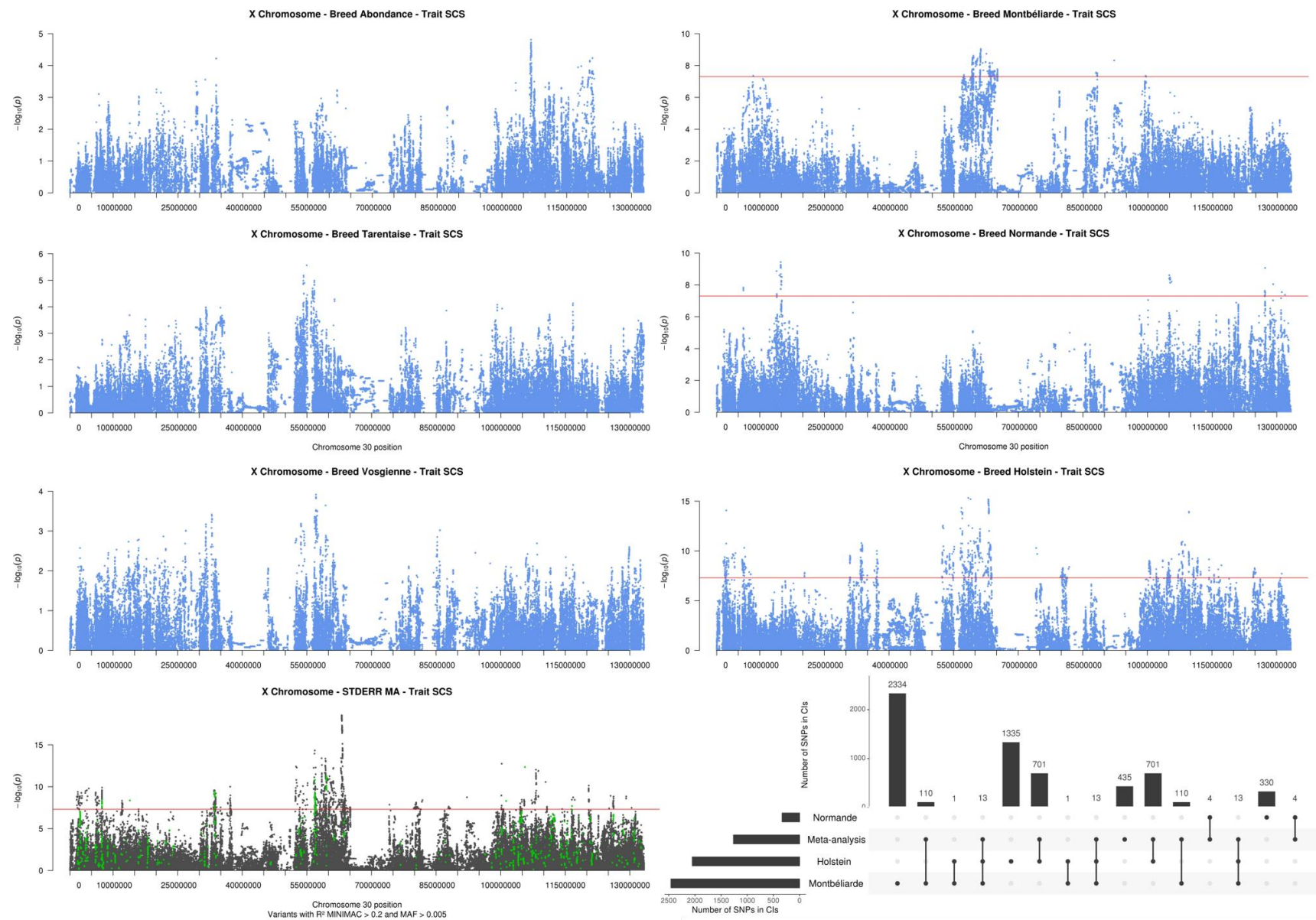

g)

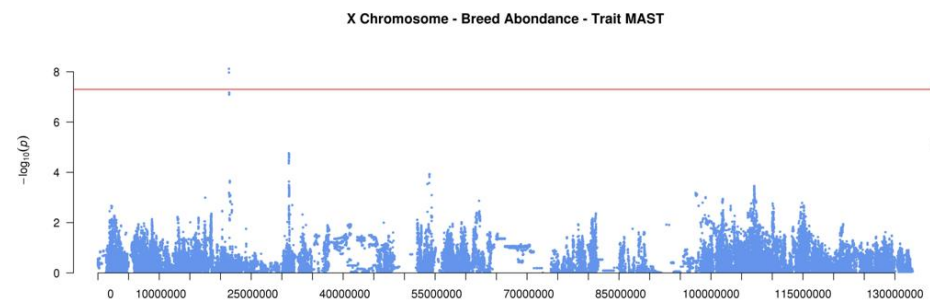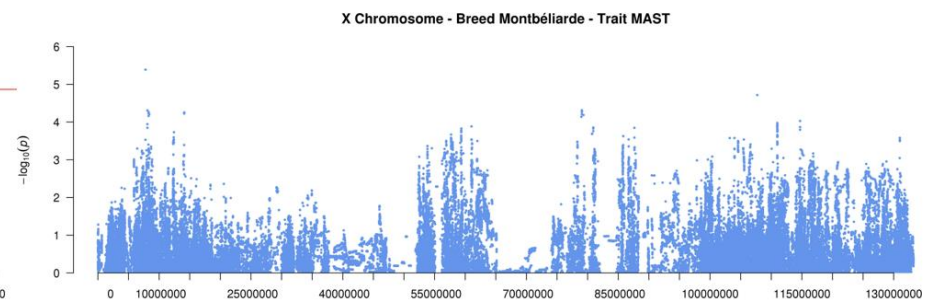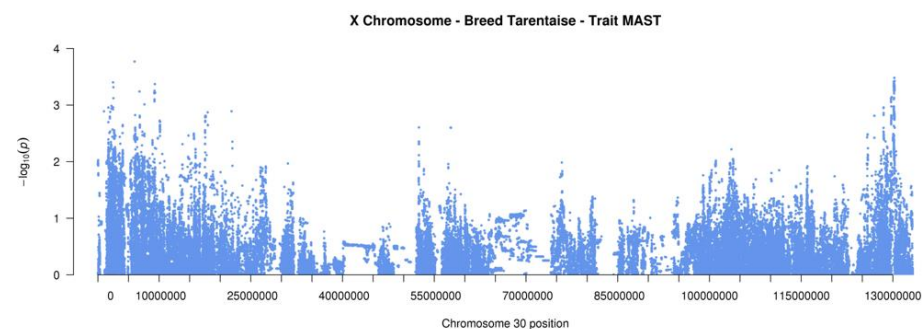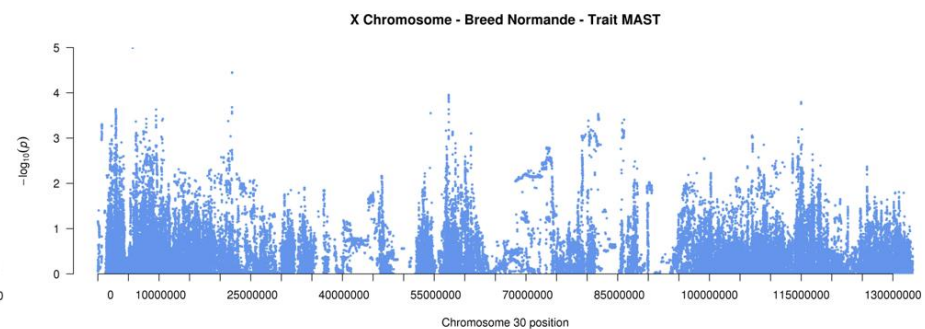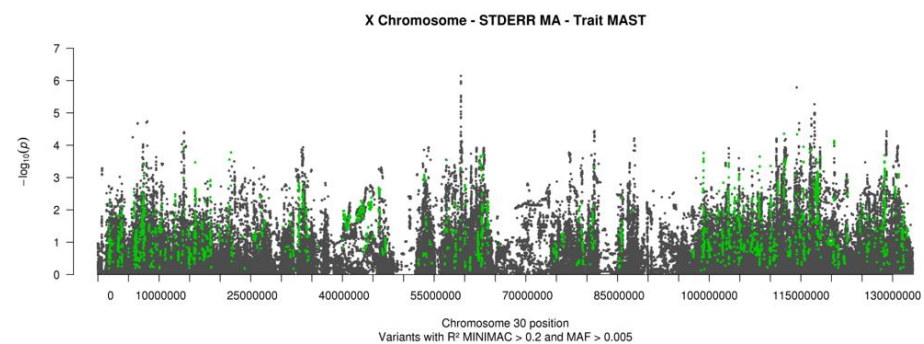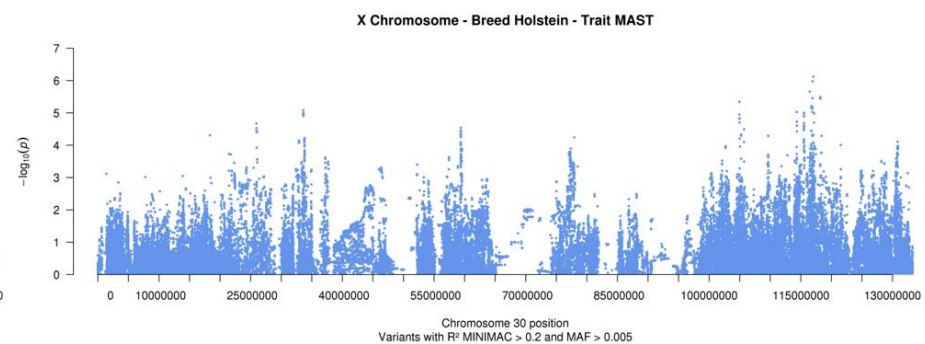

h)

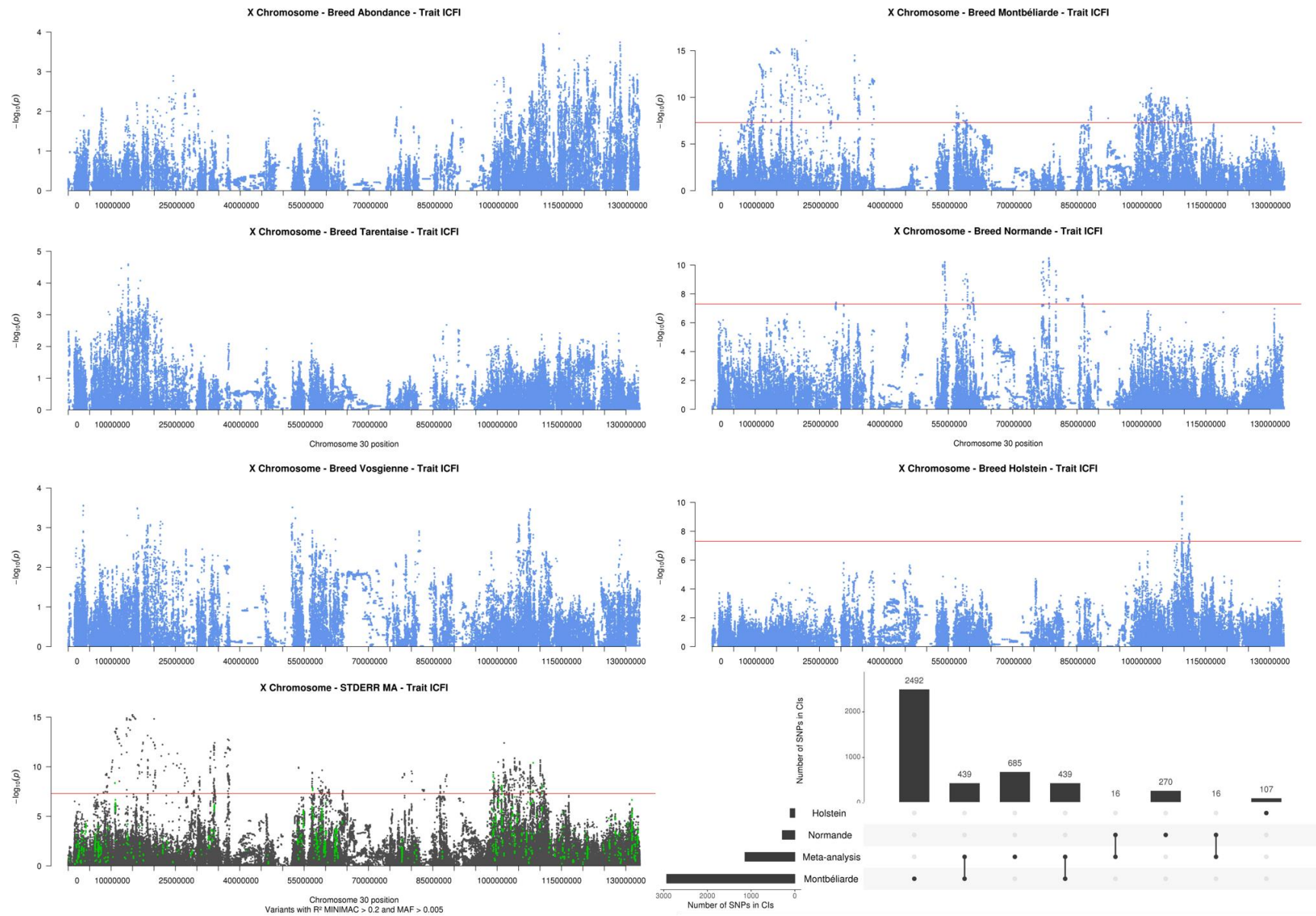

i)

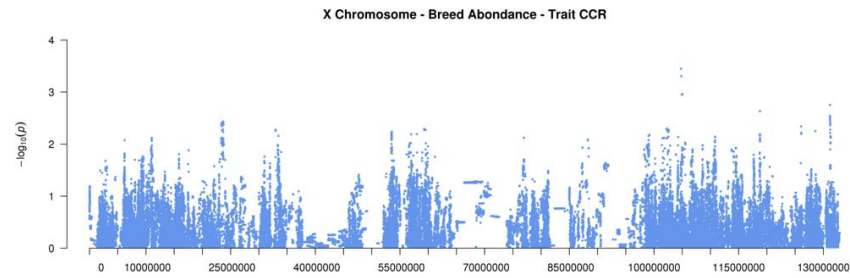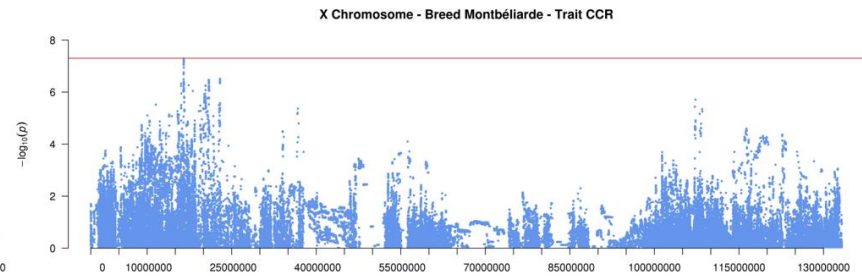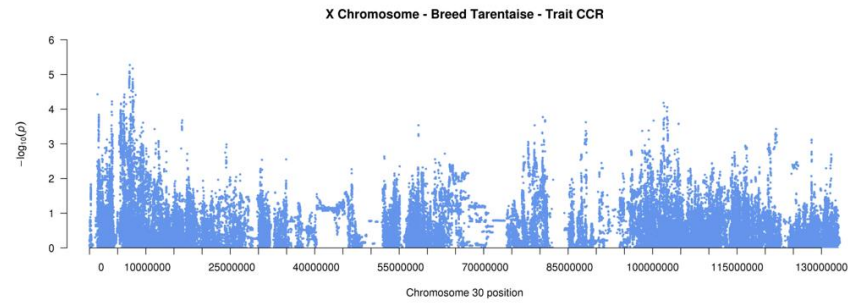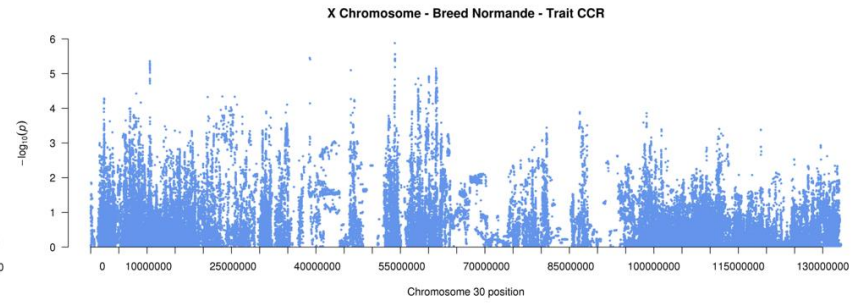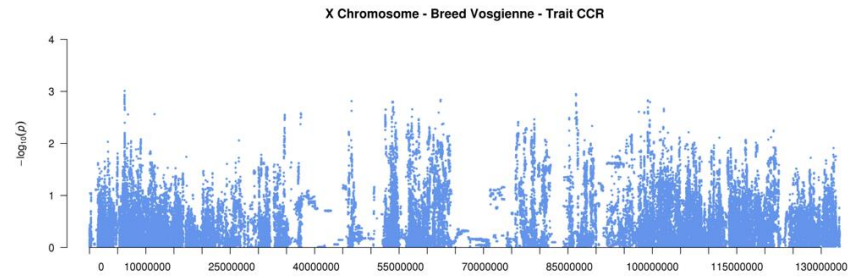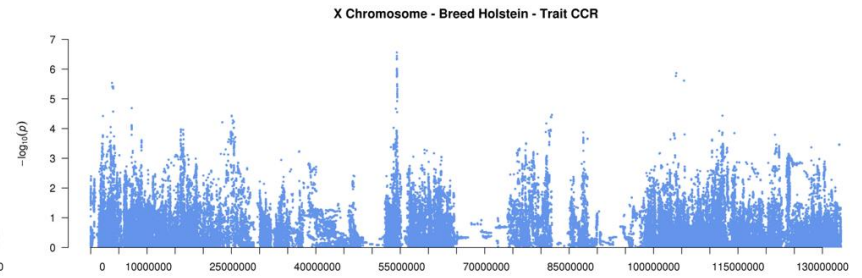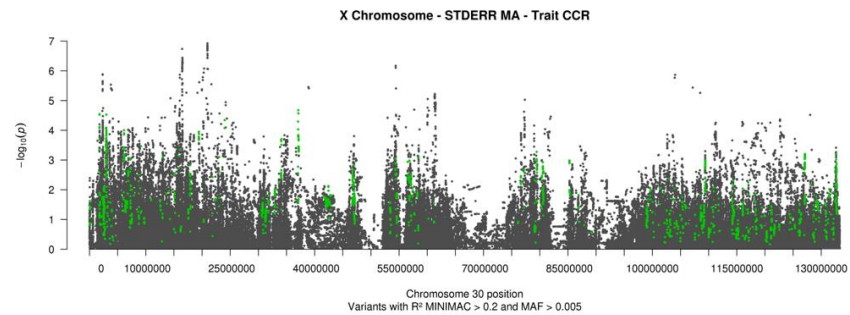

Chromosome 30 position  
Variants with  $R^2$  MINIMAC > 0.2 and MAF > 0.005

j)

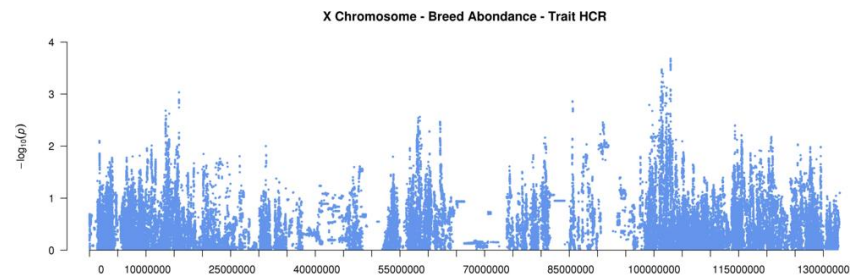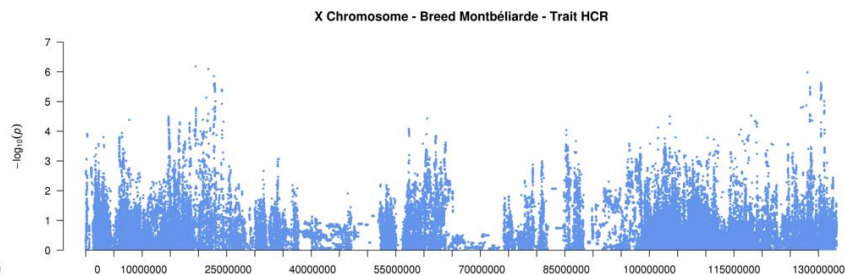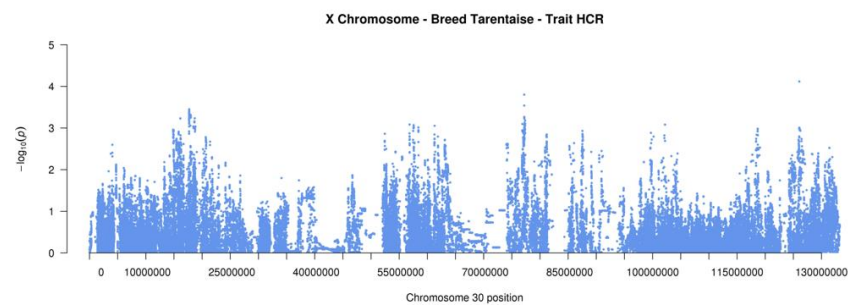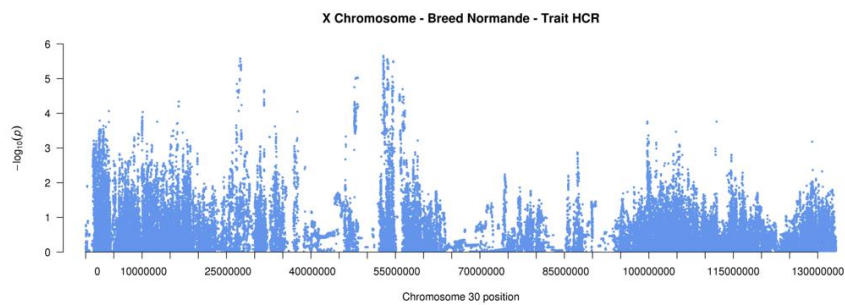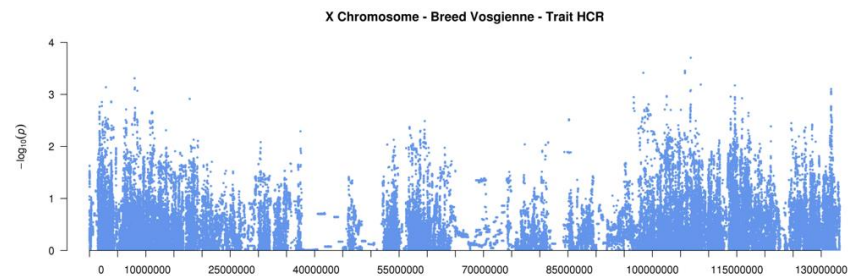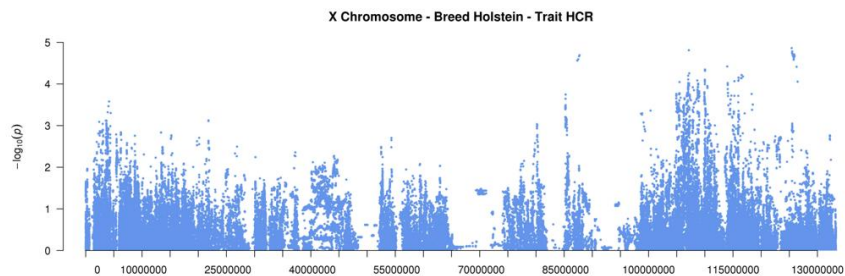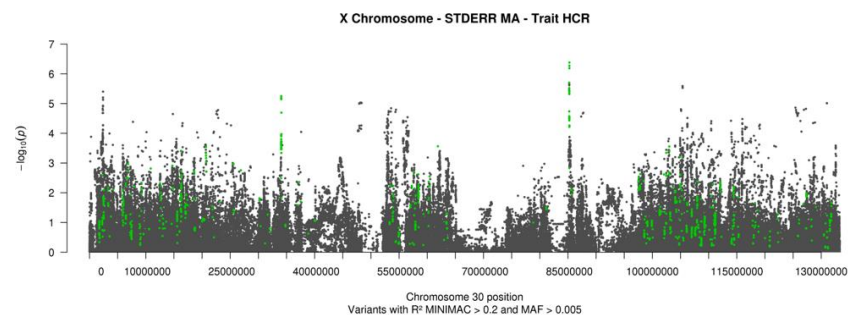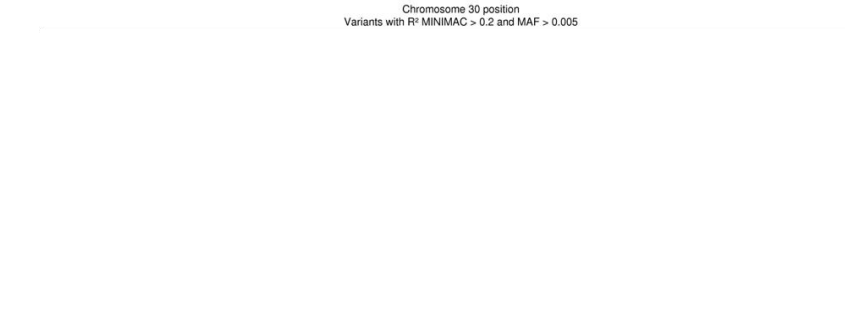

**k)**

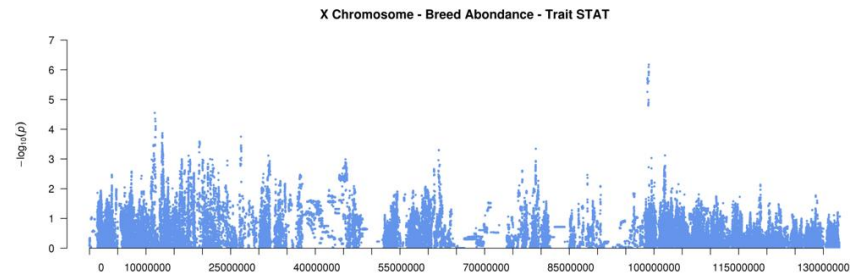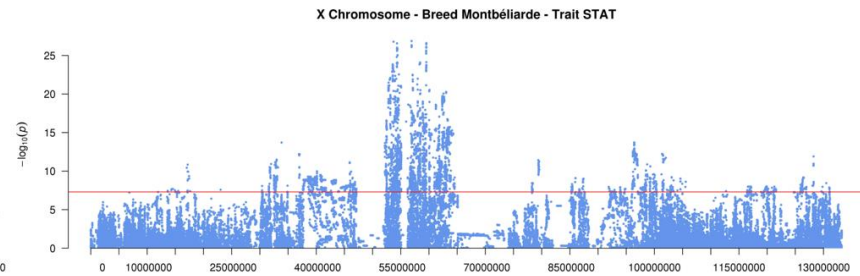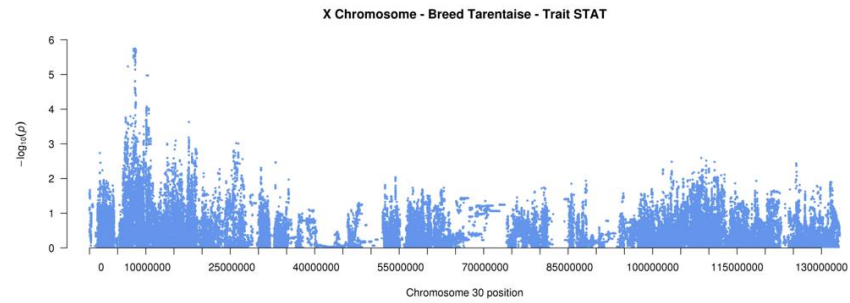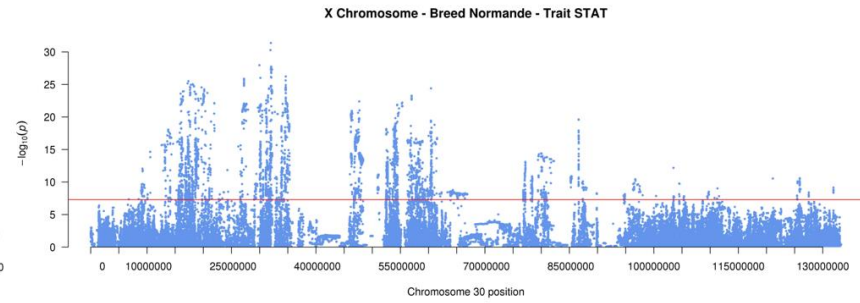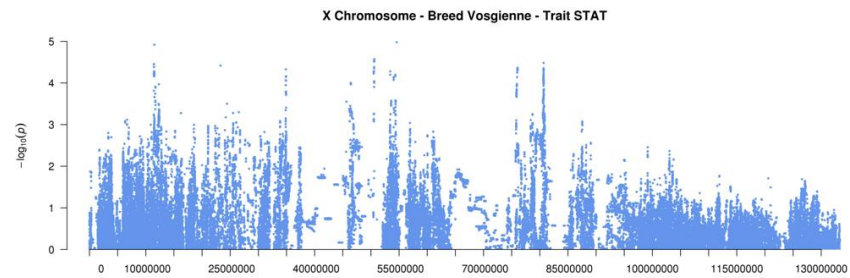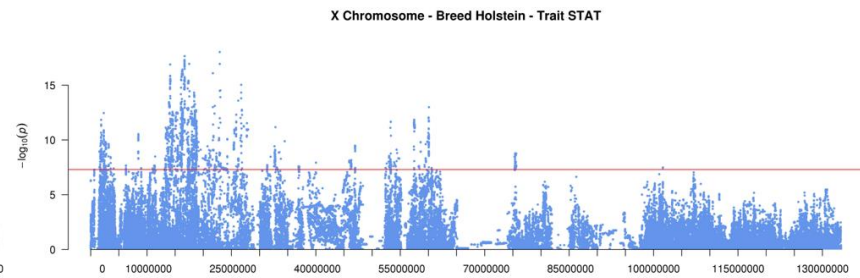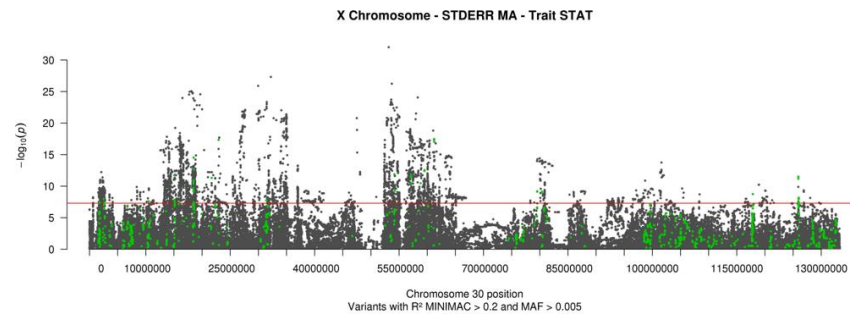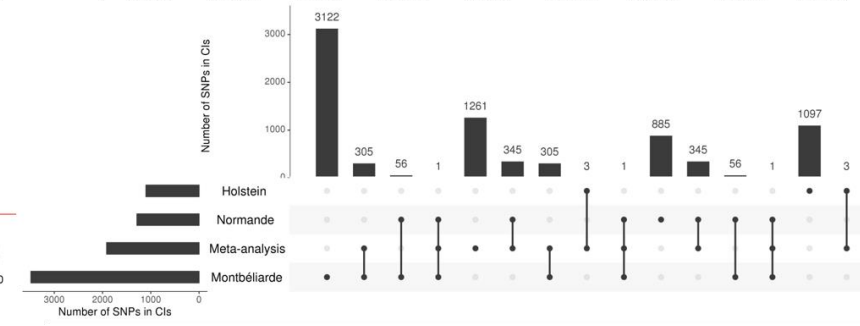

Supplement: Supplementary file 2 — Supplementary Material 2 [file 12864_2023_9438_MOESM2_ESM.pdf]

a)

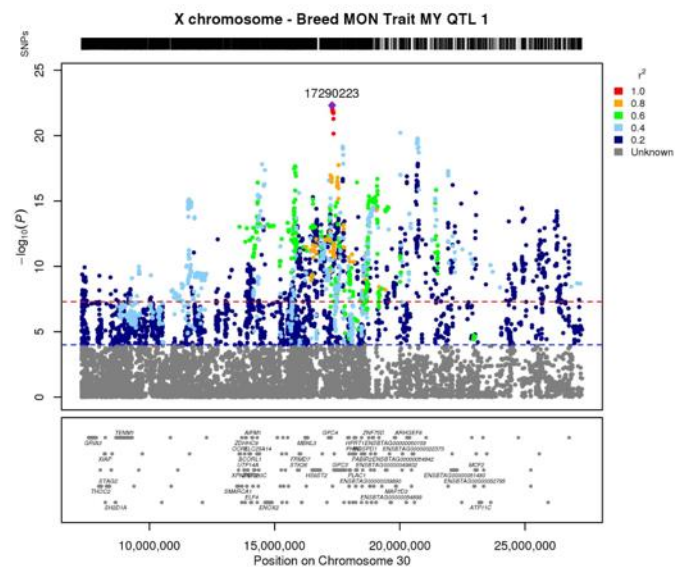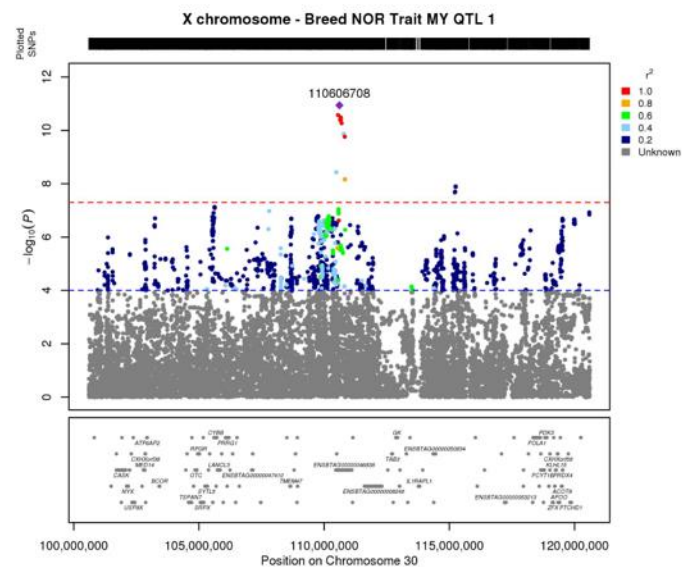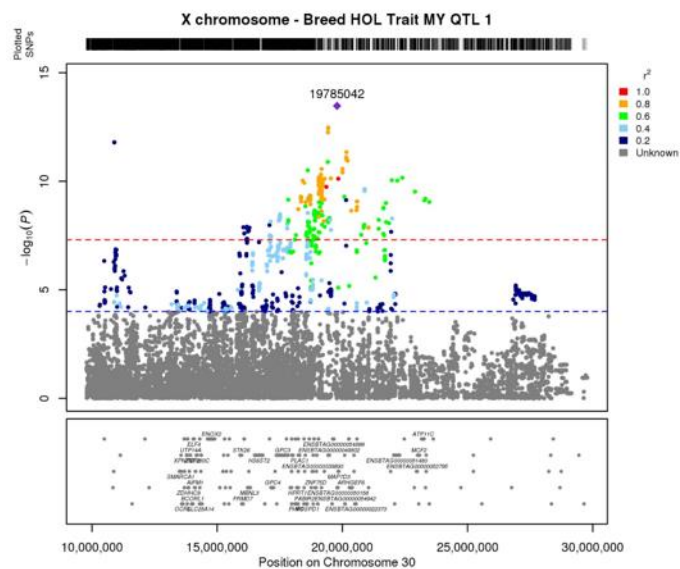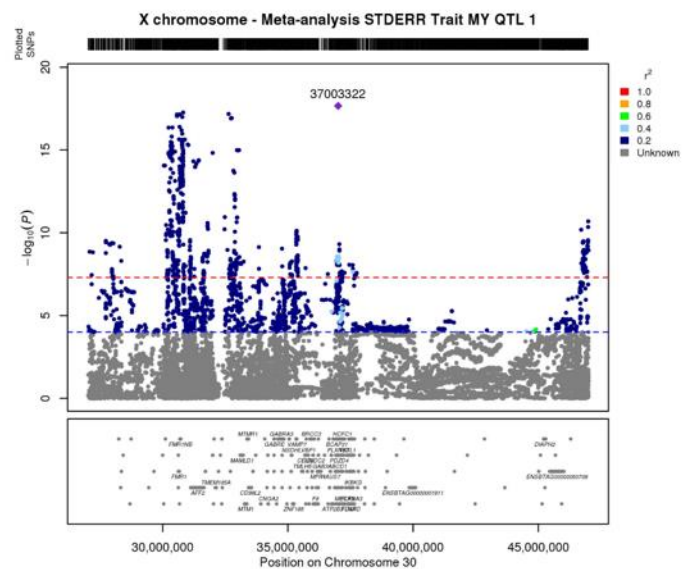

**b)**

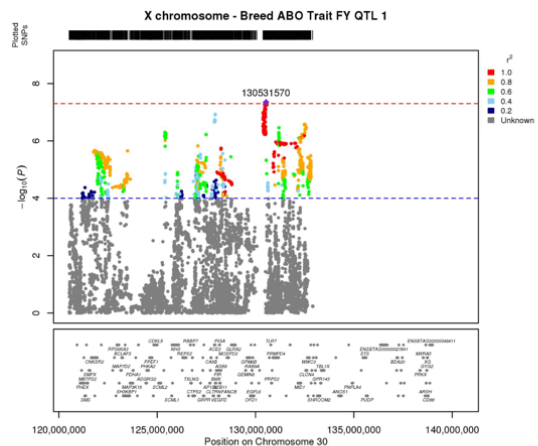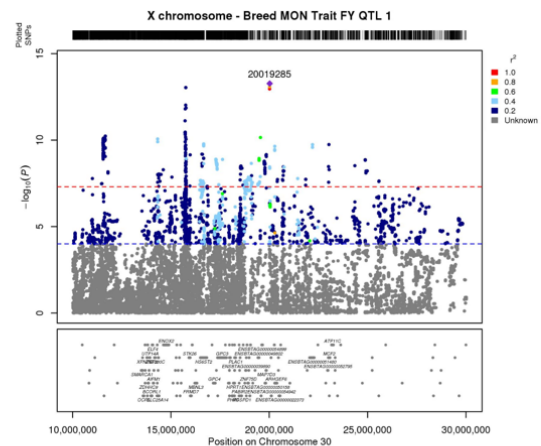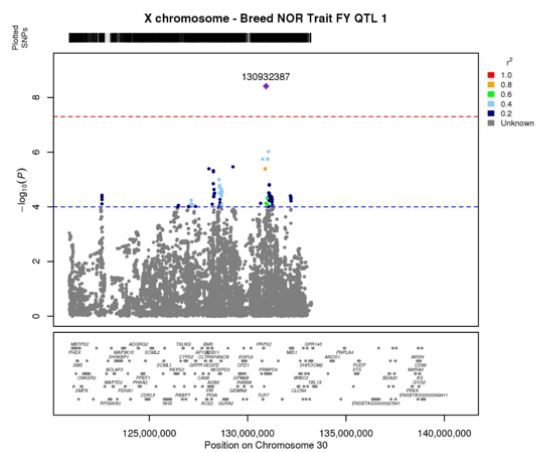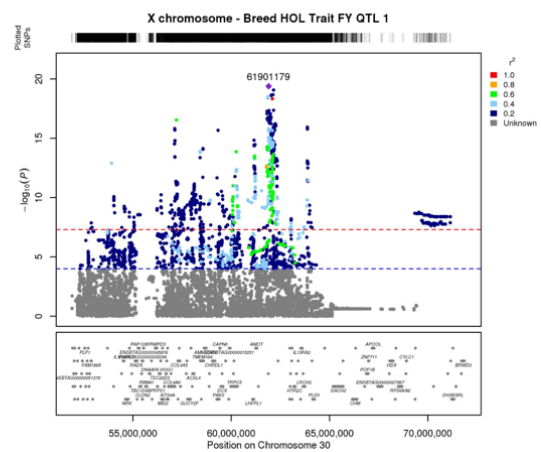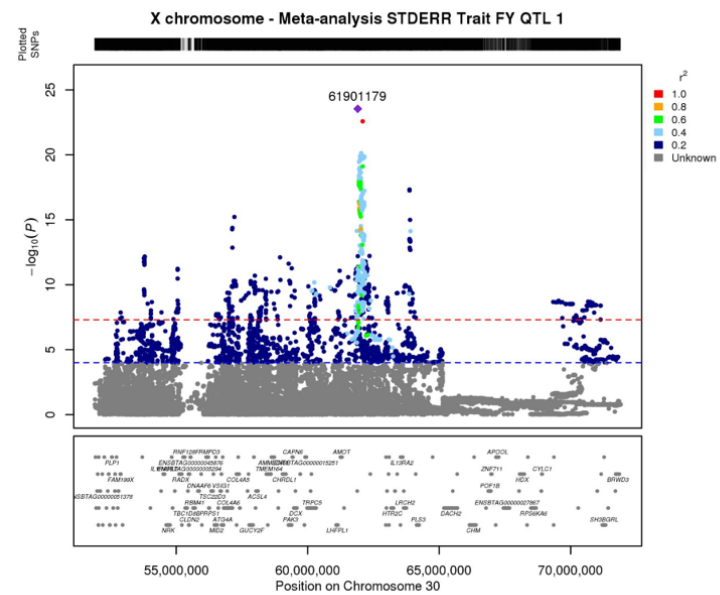

c)

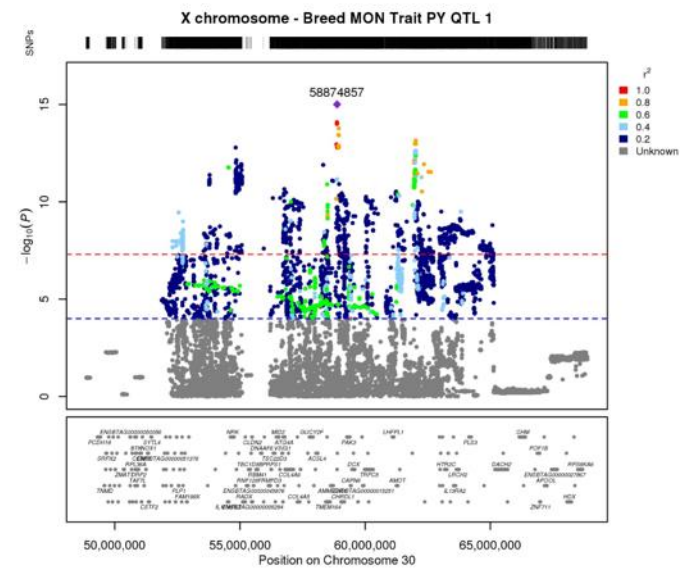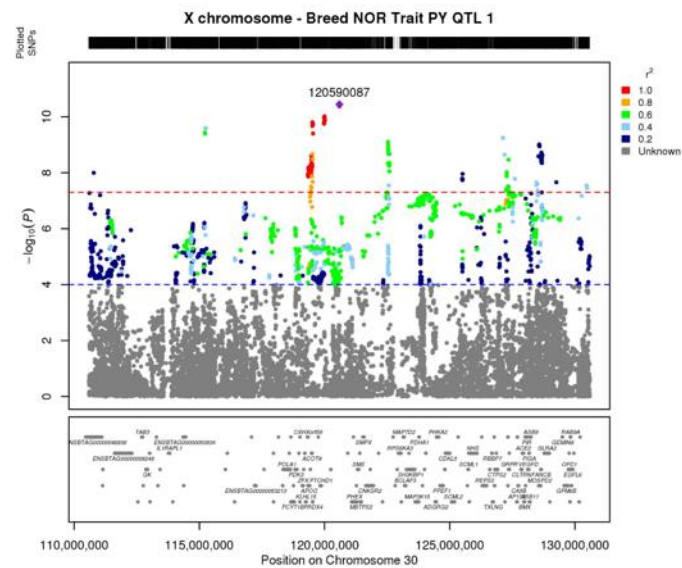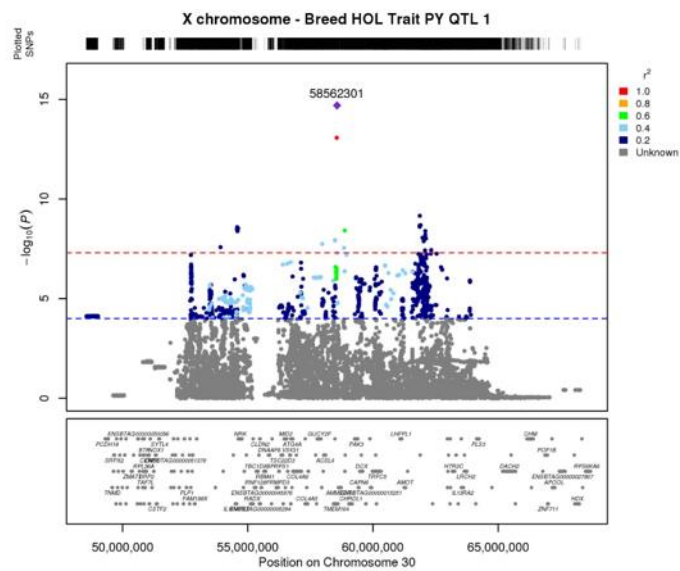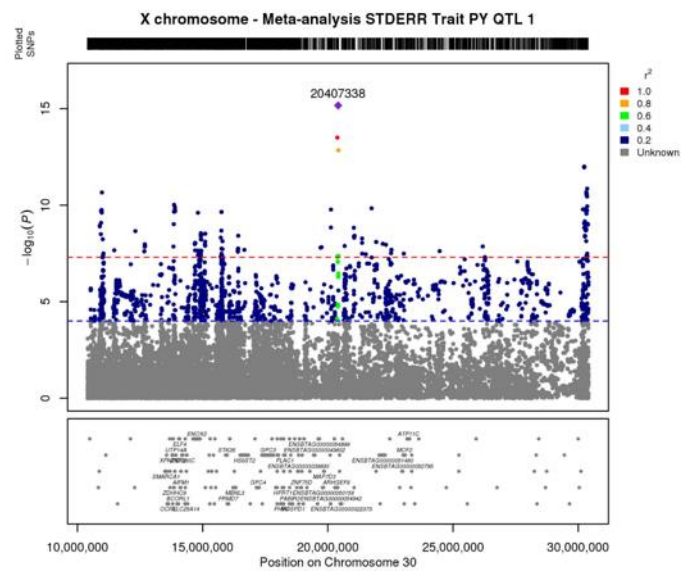

d)

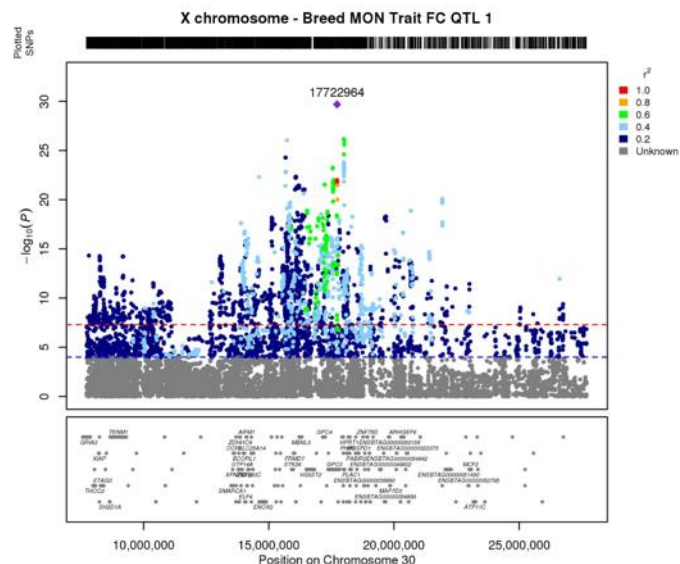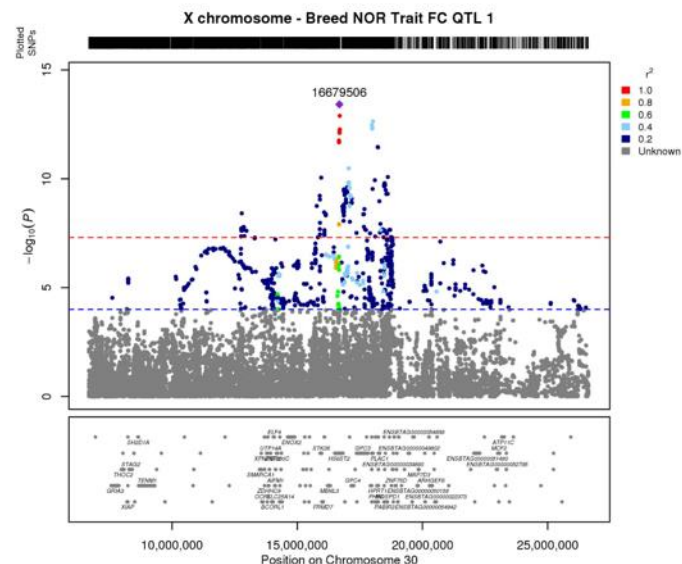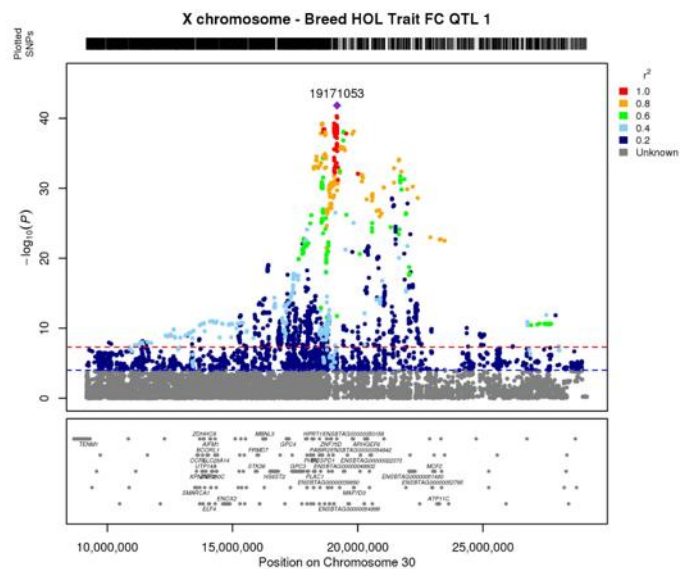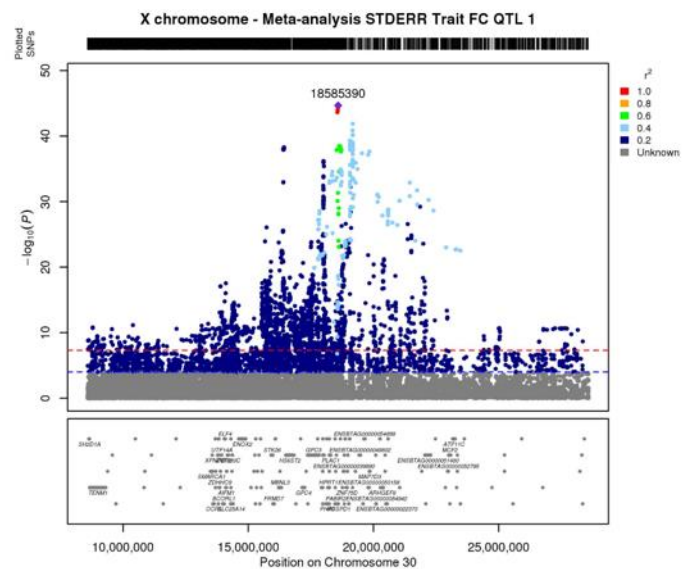

**e)**

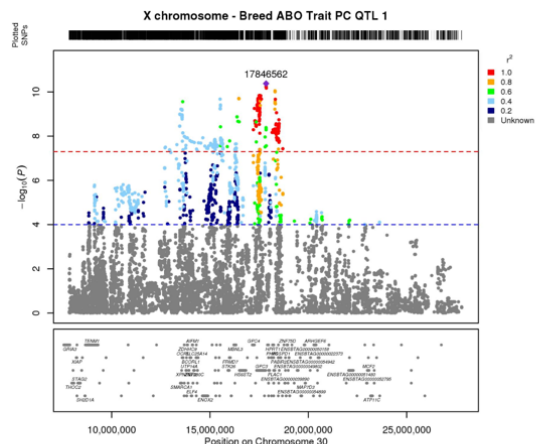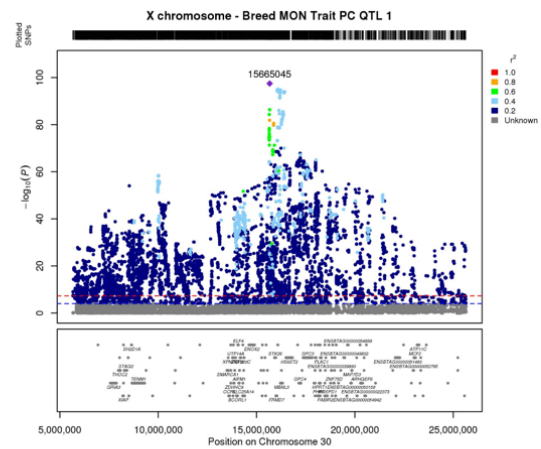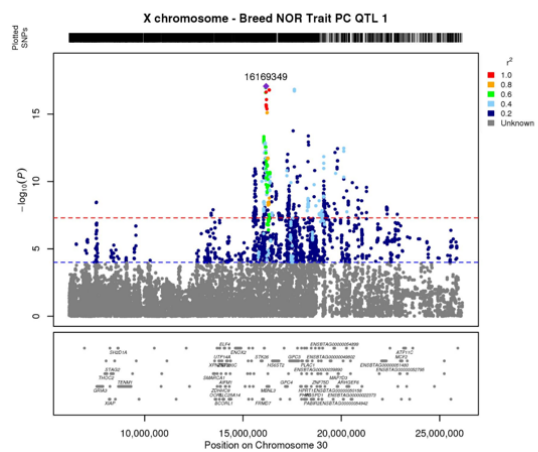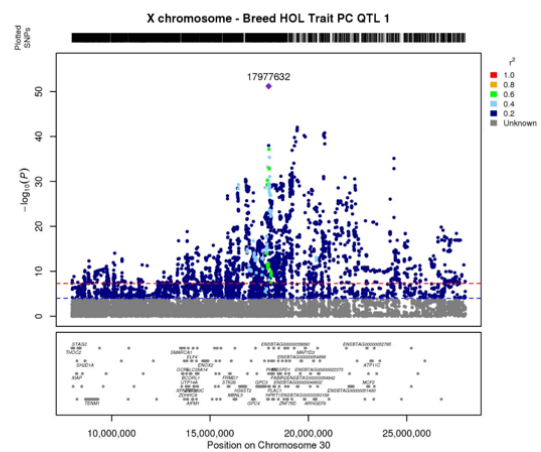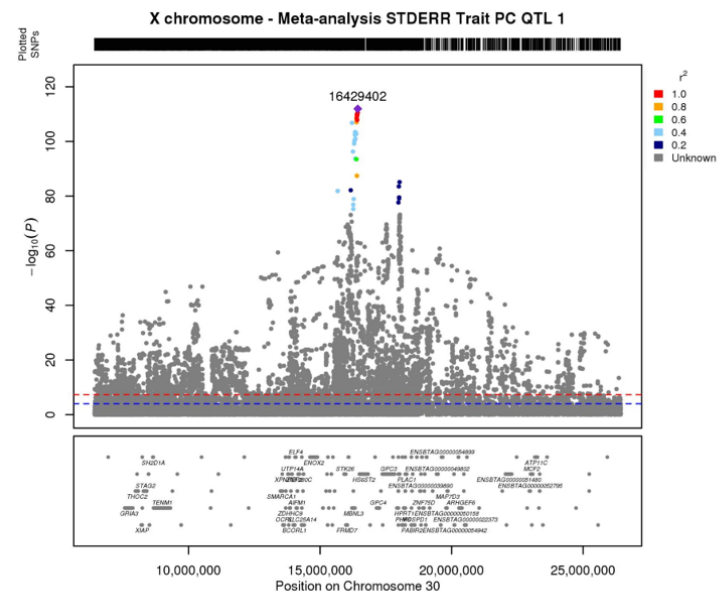

f)

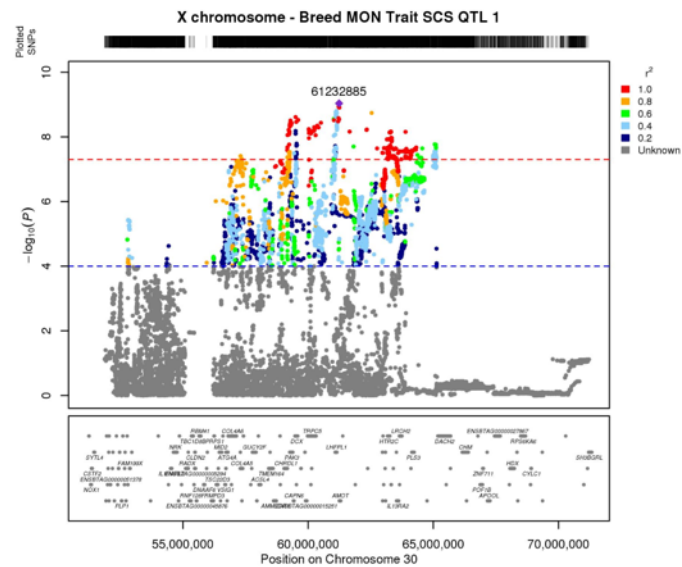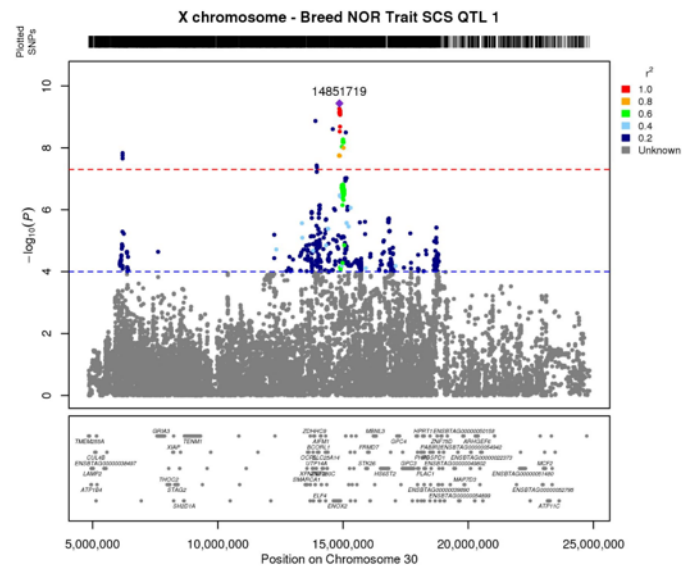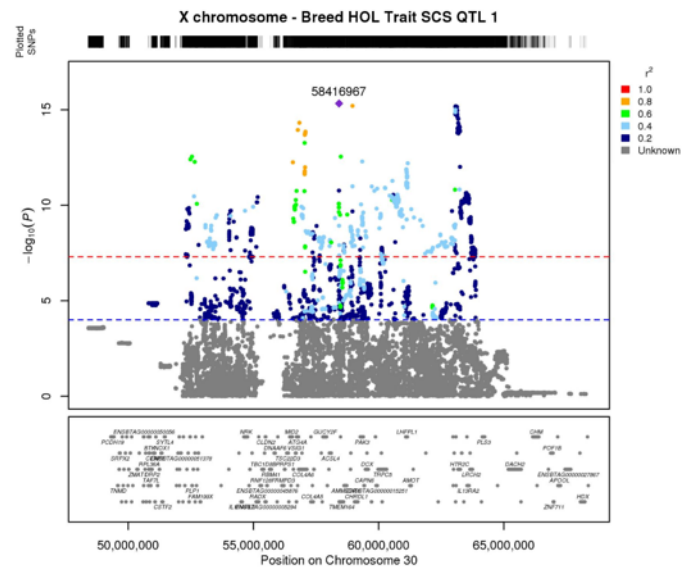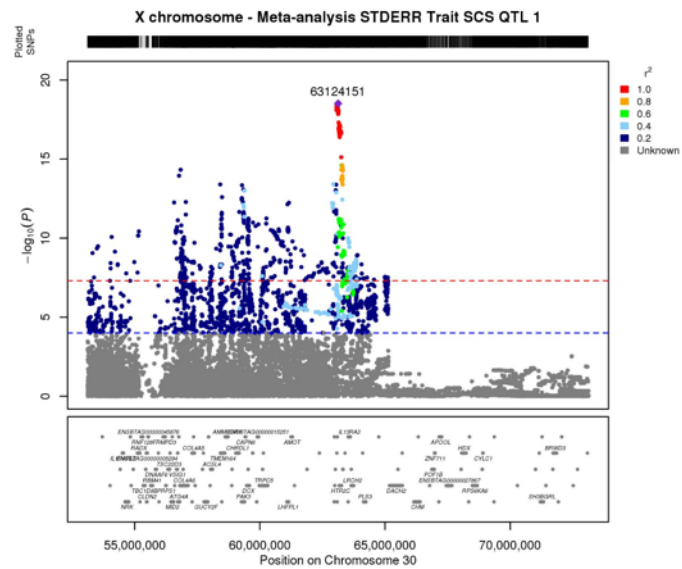

g)

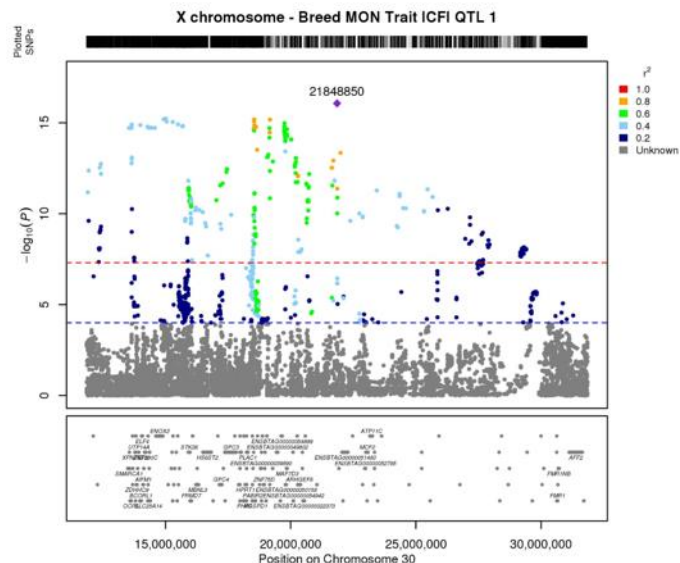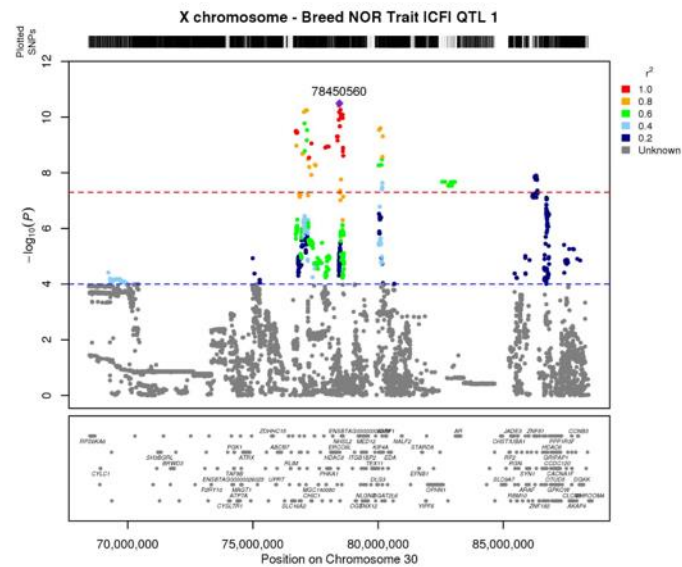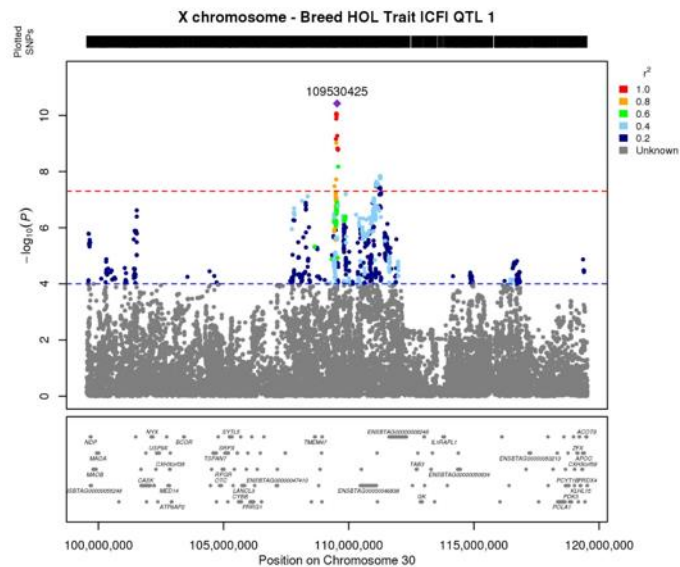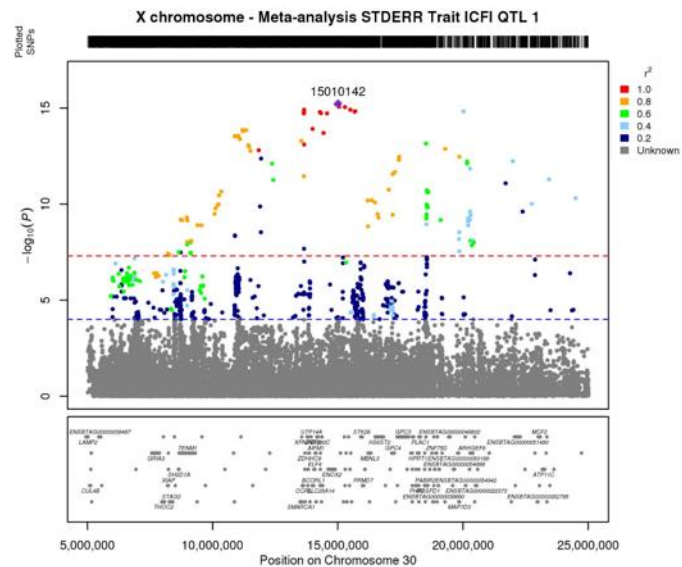

h)

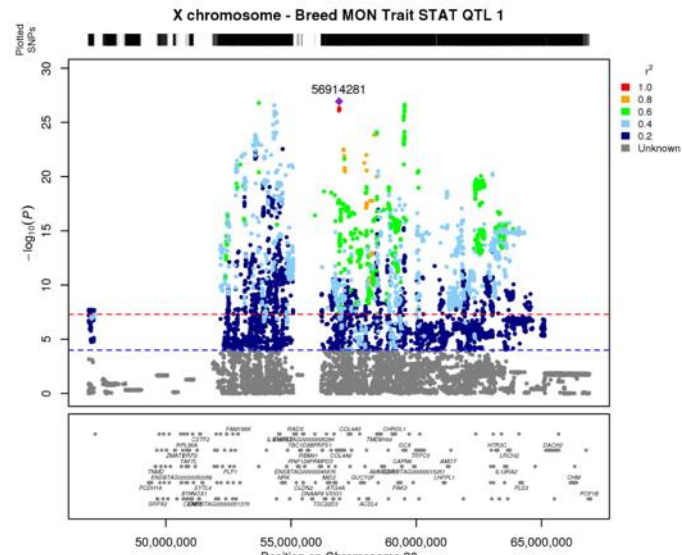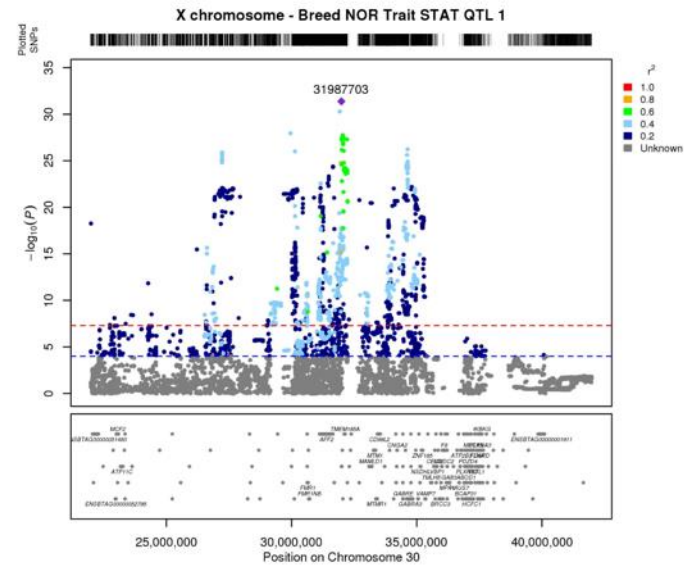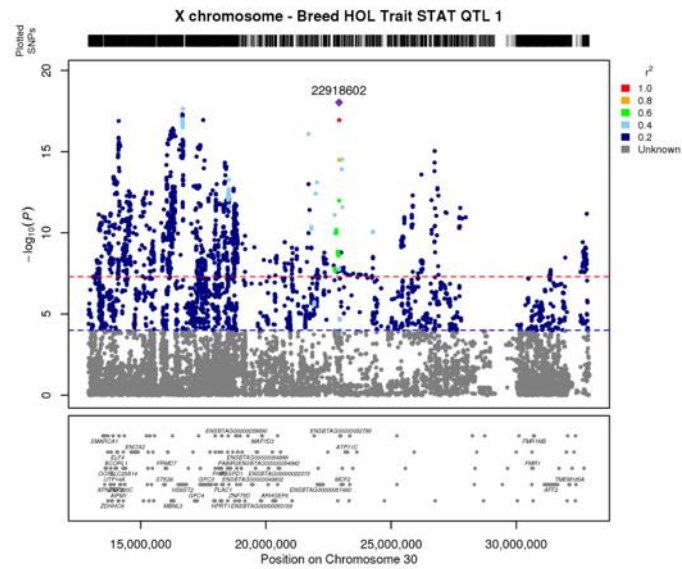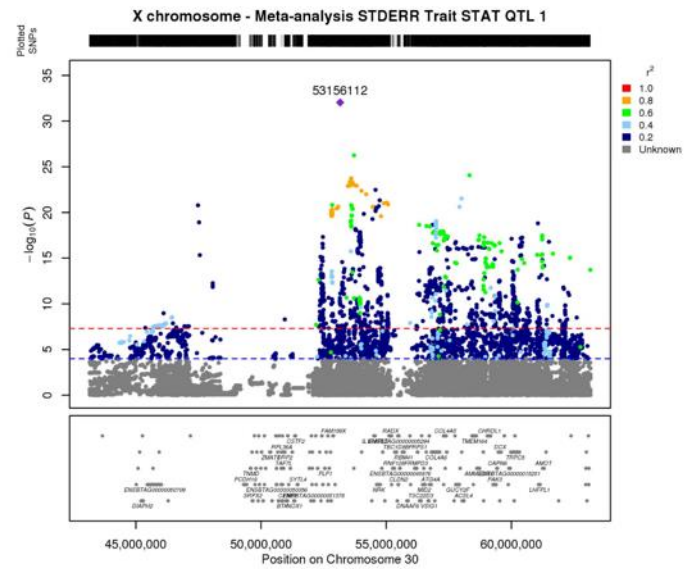

Supplement: Supplementary file 5 — Supplementary Material 5 [file 12864_2023_9438_MOESM5_ESM.pdf]
