# Supplementary material for: X-linked genes influence various complex traits in dairy cattle
Source: BMC Genomics. 2023 Jun 19;24:338. doi: 10.1186/s12864-023-09438-7 (PMC10278306; doi:10.1186/s12864-023-09438-7)
Supplement: Supplementary file 3 — Supplementary Material 3 [file 12864_2023_9438_MOESM3_ESM.docx]

# Supporting information

**S1 Table.** Reference population used to impute genotypes at the sequence level. From the 8^th^ RUN of the 1000 Bull Genome population. In bold, breeds analyzed in the present study.

S2 Table. Features of QTL identified in within-breed association analyses.

S3 Table. Features of QTL identified in meta-analyses of association.

S1 Fig. Linkage disequilibrium (r²) for the chromosomes X and 2 in the six breeds. Linkage disequilibrium was evaluated by calculating the average r² between pairs of HD SNPs within distance distance classes: 25 kb [0-200kb], 100 kb ]200-1000kb], 1000 ]1000-10 000 kb], and 5000 kb ]10 000-50 000 kb].

S2 Fig. Results of within-breed and meta-analyses of the X chromosome for each trait: Manhattan plot and number of overlapping variants located within confidence intervals of the QTL. Within-breed association analyses in Abondance (ABO), Tarentaise (TAR), Vosgienne (VOS), Montbéliarde (MON), Normande (NOR), and Holstein (HOL) cows (Manhattan plot in blue); fixed effects meta-analyses (Manhattan plot in gray, variants with effects in the same direction in all within-breed analyses are highlighted in green); and, when the same variants were identified in different analyses, UpSet diagram showing overlapping variants within confidence intervals (CIs) of the QTL; for a) milk yield (MY), b) fat yield (FY), c) protein yield (PY), d) fat content (FC), e) protein content (PC), f) somatic cell score (SCS), g) clinical mastitis (MAST), h) interval between calving and first insemination (ICFI), i) heifers’ conception rate (HCR), j) cows’ conception rate (HCR), and k) stature (STAT).

S3 Fig. LocusZoom graph of the 20-Mb region around the lead variant of the QTL with the most significant effect for each trait in within-breed and meta-analyses of the X chromosome. Within-breed association analyses in Abondance (ABO), Montbéliarde (MON), Normande (NOR), and Holstein (HOL) cows, together with fixed effects meta-analyses (STDERR), for a) milk yield (MY), b) fat yield (FY), c) protein yield (PY), d) fat content (FC), e) protein content (PC), f) somatic cell score (SCS), interval between calving and first insemination (ICFI), and g) stature (STAT)
